# AAV vector-mediated *ALDH5A1* gene replacement improves survival and metabolic correction in a severe mouse model of SSADH deficiency

**DOI:** 10.1016/j.omta.2026.201860

**Published:** 2026-10-07

**Authors:** Kiri Koshu, Ayumi Horiguchi, Teru Mizuno, Shiho Aoki, Miyuki Watanabe, Naomi Takino, Mika Ito, Eriko F. Jimbo, Kazuhiro Muramatsu, Shin-Ichi Muramatsu, Hitoshi Osaka

**Affiliations:** 1 Department of Pediatrics, Jichi Medical University, Shimotsuke, Tochigi 329-0498, Japan; 2 Division of Neurological Gene Therapy, Center for Open Innovation, Jichi Medical University, Shimotsuke, Tochigi 329-0498, Japan

**Keywords:** succinic semialdehyde dehydrogenase deficiency, SSADH deficiency, aldehyde dehydrogenase 5 family member A1, *ALDH5A1*, γ-aminobutyric acid, GABA, γ-hydroxybutyrate, GHB, seizures, liquid chromatography-tandem mass spectrometry, LC-MS/MS, quantification, gene therapy, adeno-associated virus, vector, AAV vector, intracisternal injection

## Abstract

Succinic semialdehyde dehydrogenase (SSADH) deficiency is a severe neurometabolic disorder characterized by γ-aminobutyric acid (GABA) and γ-hydroxybutyrate (GHB) accumulation, causing network hyperexcitability, progressive neuroglial pathology, and premature death. Currently, no curative therapy is available. We developed an adeno-associated virus (AAV) vector expressing human aldehyde dehydrogenase 5 family member A1 and assessed its therapeutic efficacy in a severe SSADH-deficient mouse model following neonatal administration at escalating doses. AAV vector-mediated gene replacement conferred therapeutic benefit, including extended survival, reduced mortality, enhanced motor performance, and significant reductions in urinary GABA and GHB levels. The survival benefit was most evident at the high dose, suggesting the presence of an apparent therapeutic threshold for survival benefit. Vector genomes were distributed throughout the central nervous system and liver, with the robust restoration of SSADH expression in the central nervous system. These findings provide strong preclinical justification for the clinical translation of central nervous system-directed AAV vector gene therapy in this disorder.

## Graphical abstract

**Figure:**
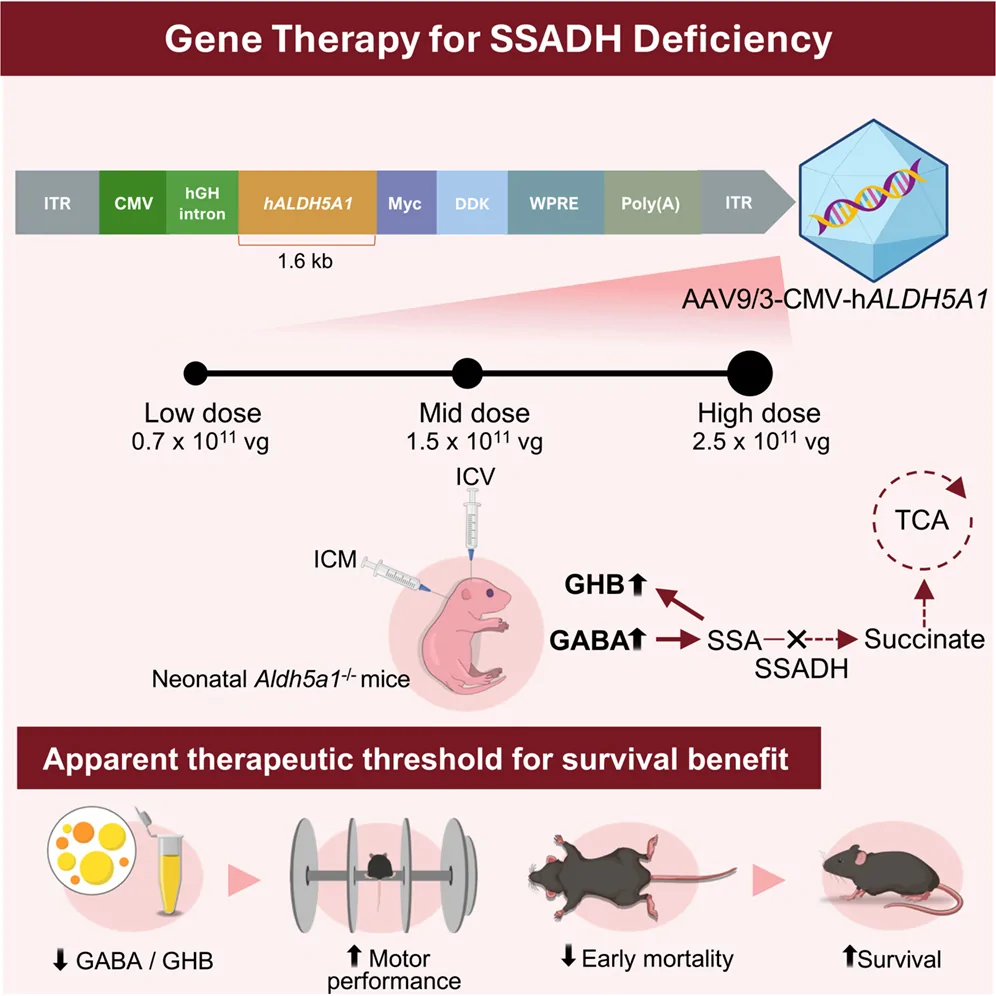

## Teaser

AAV-mediated *ALDH5A1* gene replacement improves survival, motor performance, and urinary GABA/GHB abnormalities in a severe SSADH-deficient mouse model. These findings suggest an apparent therapeutic threshold for survival benefit and support further development of CNS-directed AAV vector gene therapy for SSADH deficiency.

## Introduction

Succinic semialdehyde dehydrogenase deficiency (SSADH deficiency; OMIM: 271980) is a rare autosomal recessive inborn error of metabolism caused by loss-of-function mutations in the aldehyde dehydrogenase 5 family member A1 (*ALDH5A1*) gene on chromosome 6p22.^1^^,^^2^ Dysfunction of SSADH results in the accumulation of γ-aminobutyric acid (GABA) and γ-hydroxybutyrate (GHB), in the brain and body fluids (Figure 1A). The disorder predominantly affects the central nervous system (CNS). Affected individuals present in infancy with hypotonia and developmental delay, and subsequently develop ataxia and intellectual disability; around half of patients also develop epilepsy, and sudden unexpected death in epilepsy has been reported in up to 12% of affected individuals.^3^^,^^4^^,^^5^^,^^6^ This substantial risk of epilepsy-related mortality highlights the need for disease-modifying therapies that improve both neurological outcomes and survival. The worldwide prevalence is estimated at approximately 1 in 460,000.^7^ SSADH deficiency leads to prenatal accumulation of GABA and GHB, disrupting neuronal network maturation and altering inhibitory neurotransmission during early brain development.^8^^,^^9^^,^^10^^,^^11^ Diagnosis is suggested by elevated GHB levels in urine organic acid analysis and confirmed by genetic testing; however, diagnosis is often delayed because of its rarity and phenotypic heterogeneity.^5^

**Figure 1:**
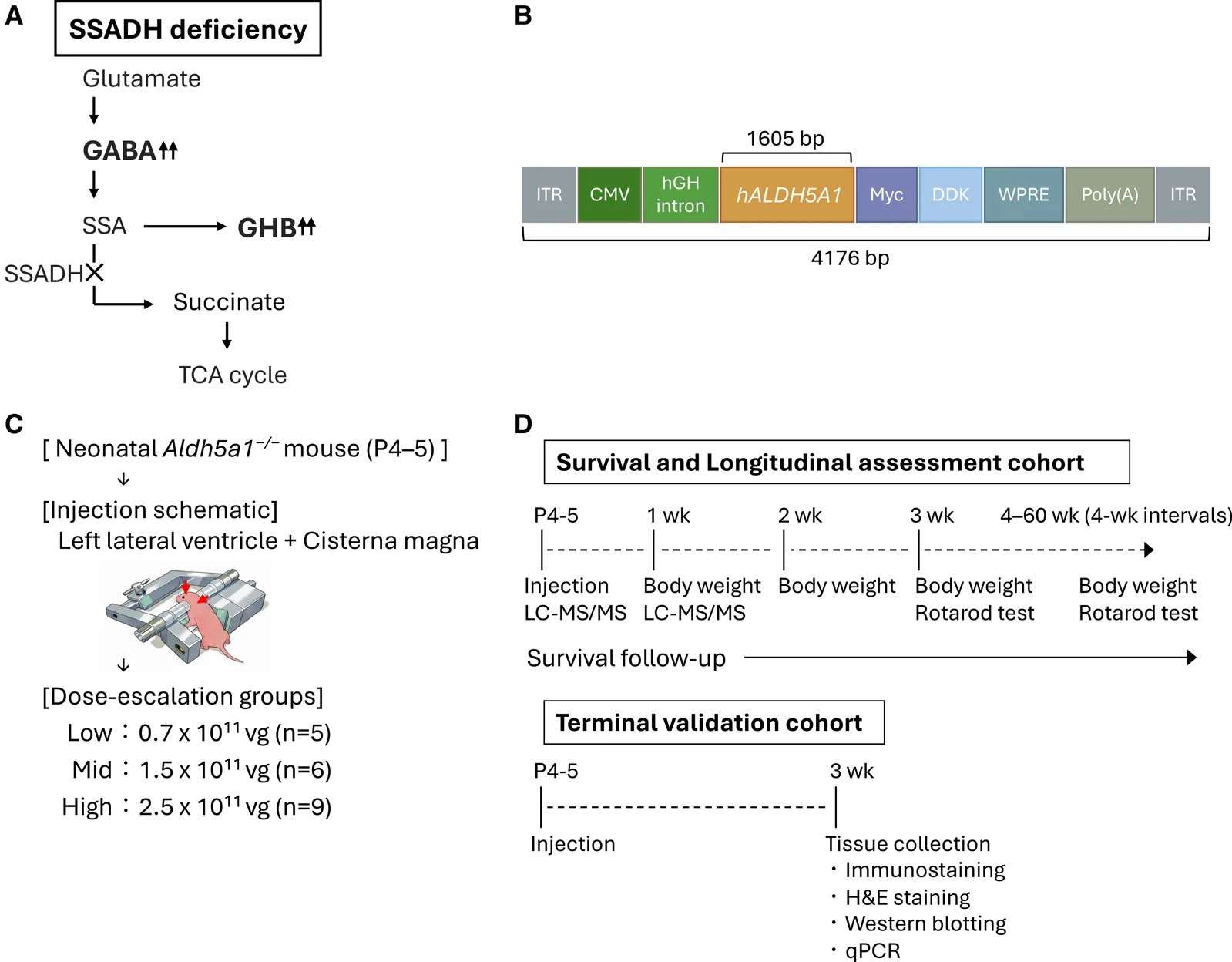
Study design and adeno-associated virus-human aldehyde dehydrogenase 5 family member A1 vector gene therapy strategy (A) Schematic representation of γ-aminobutyric acid (GABA) metabolism and pathophysiology of succinic semialdehyde dehydrogenase (SSADH) deficiency. Loss of SSADH impairs the conversion of succinic semialdehyde to succinate, resulting in the accumulation of GABA and γ-hydroxybutyric acid and disruption of the tricarboxylic acid cycle. (B) Structure of the adeno-associated virus (AAV) 9/3-cytomegalovirus (CMV)-human aldehyde dehydrogenase 5 family member A1 (h-*ALDH5A1*) expression cassette. The vector contains inverted terminal repeats (ITRs), a CMV promoter, a human growth hormone (hGH) intron, the human-*ALDH5A1* coding sequence (approximately 1.6 kb), Myc and DYKDDDK(DDK) epitope tags, the woodchuck hepatitis virus post-transcriptional regulatory element (WPRE), and a polyadenylation [poly(A)] signal. (C) Experimental design for dose escalation. Neonatal *Aldh5a1*^−/−^ mice (postnatal days 4–5 [P4-5]) received sequential intracerebroventricular and cisterna magna injections of AAV-h*ALDH5A1* vector at low (0.7 × 10^11^ vector genomes [vg]), mid (1.5 × 10^11^ vg), or high (2.5 × 10^11^ vg) doses. (D) Study timeline. The AAV vector was administered on postnatal days 4–5 (P4-5). Liquid chromatography-tandem mass spectrometry (LC-MS/MS) analysis was performed at the time of injection (P4-5) and at 1 week to assess early biochemical alterations. Body weight was assessed weekly from 1 to 4 weeks of age and every 4 weeks thereafter from 8 to 60 weeks of age, with the exception of the low-dose group, for which measurements were not obtained at weeks 1 and 2 and only one measurement was available at week 4. Rotarod performance was assessed at 3 and 4 weeks of age and every 4 weeks thereafter from 8 to 60 weeks of age. Molecular and histological analyses (immunostaining, hematoxylin and eosin [H&E] staining, western blotting, and quantitative polymerase chain reaction [qPCR]) were conducted at 3 weeks of age. Survival was monitored until death or the end of the observation period. Animals used for LC-MS/MS analysis partially overlapped with the survival and longitudinal assessment cohort, whereas separate cohorts were used for molecular and histological analyses at 3 weeks of age.

Currently, no curative therapy is available. Because this disorder results from ALDH5A1 deficiency, restoring its activity represents a rational strategy to directly correct the underlying biochemical defect. Additionally, the relatively small size of *ALDH5A1* cDNA (1.6 kb) enables efficient packaging into an adeno-associated virus (AAV) vector,^12^ thereby enabling CNS-directed gene replacement. This supports the feasibility of gene therapy as a promising therapeutic strategy.^2^^,^^13^ *Aldh5a1*^−/−^ mice closely recapitulate the human SSADH deficiency phenotype, demonstrating increased GABA and GHB levels in tissues and urine, seizures that progress by 3–4 weeks of age, and early death.^14^^,^^15^^,^^16^^,^^17^ Liver-directed adenoviral vectors administered intraperitoneally or retro-orbitally demonstrated partial and transient reductions in GHB levels, and modestly extended survival in a subset of *Aldh5a1*^−/−^ mice.^13^ Although these studies provided proof-of-concept for gene therapy in SSADH deficiency, an efficient and durable CNS-wide transgene expression remained challenging. Recent studies have further demonstrated the feasibility of AAV-mediated *ALDH5A1* gene replacement in severe SSADH mouse models.^18^ In this study, we evaluated the therapeutic efficacy of AAV-mediated *ALDH5A1* gene replacement in *Aldh5a1*^−/−^ mice (Figures 1B–1D).

## Results

### Survival analyses

Untreated *Aldh5a1*^−/−^ mice exhibited a median survival of 24 days (range, 16–81 days), whereas saline-treated mice showed a similar median survival of 27 days (Figure 2A). High-dose AAV treatment markedly prolonged survival, with a median survival of 433.5 days, significantly longer than that of untreated *Aldh5a1*^−/−^ mice (log-rank test, *p* < 0.001; hazard ratio (HR), 0.20; 95% confidence interval [CI], 0.09–0.48). No deaths occurred within the first month of life in the high-dose group. Mid-dose AAV treatment showed prolonged survival, with a median survival of 262.5 days; however, the difference was not statistically significant after Bonferroni correction (log-rank test, *p* = 0.04; HR, 0.42; 95% CI, 0.18–0.97). Notably, the mid-dose group exhibited a bimodal survival pattern, with some mice showing long-term survival, whereas others died within the first month of life. In contrast, low-dose treatment failed to alter disease progression significantly. The median survival was 22 days, and four of five mice died within the first month of life (log-rank test, *p* = 0.87). For the three pairwise comparisons of AAV-treated groups versus untreated *Aldh5a1*^−/−^ mice, statistical significance was assessed using a Bonferroni-corrected threshold of *p* < 0.0167.

**Figure 2:**
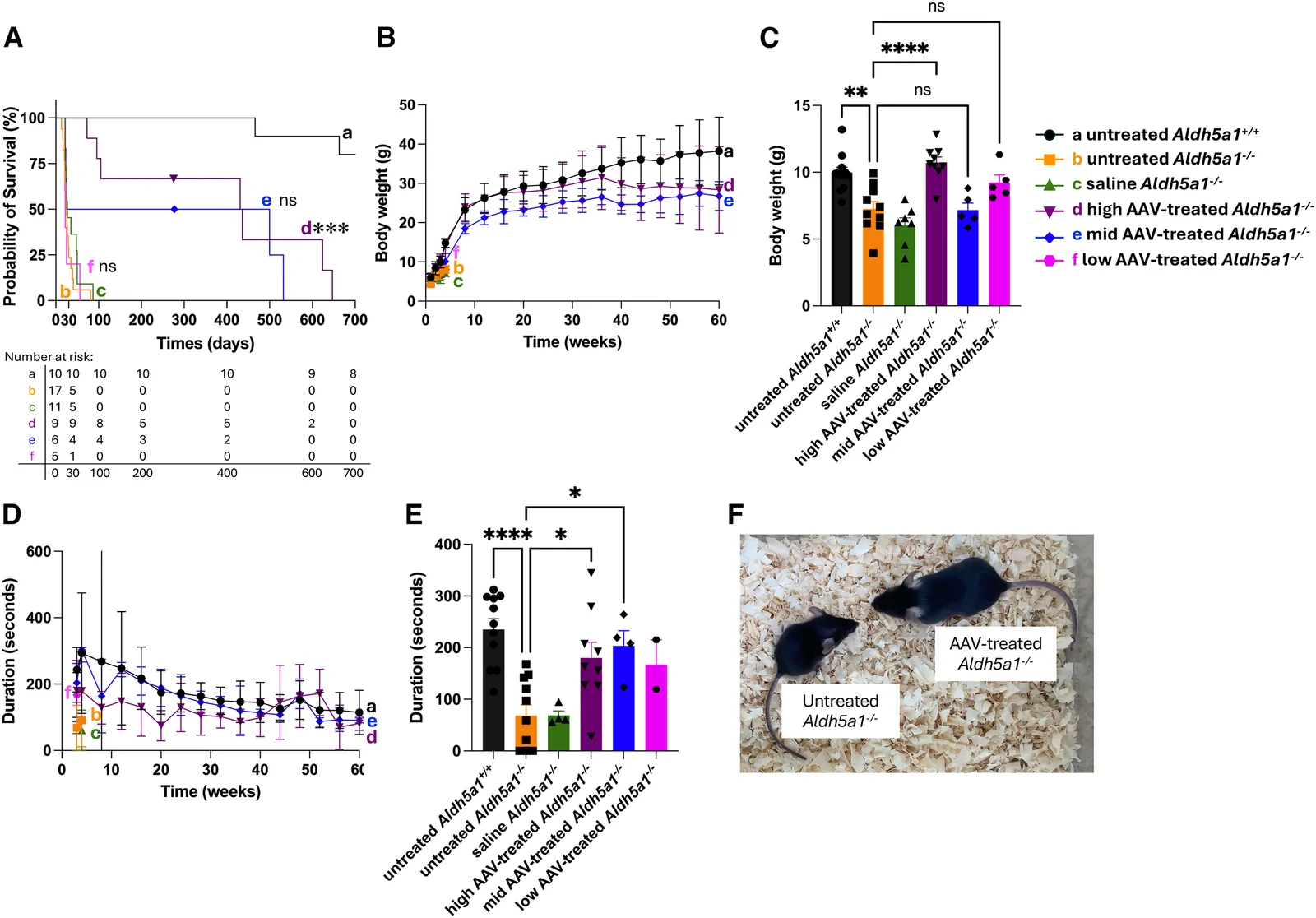
Therapeutic effects of neonatal adeno-associated virus vector-mediated gene replacement in aldehyde dehydrogenase 5 family member A1^−/−^ mice (A) Kaplan-Meier survival curves for untreated aldehyde dehydrogenase 5 family member A1 (*Aldh5a1*)^*+/+*^ mice, untreated *Aldh5a1*^−/−^ mice, saline-treated *Aldh5a1*^−/−^ mice, and adeno-associated virus (AAV)-treated *Aldh5a1*^−/−^ mice receiving high-dose, mid-dose, or low-dose vector. Untreated and saline-treated *Aldh5a1*^−/−^ mice exhibited early mortality, whereas high-dose AAV vector treatment markedly prolonged survival, with no deaths occurring within the first month of life. Mid-dose AAV treatment produced an intermediate response, with a subset of mice surviving long term while others died within the first month of life. Survival curves were compared using the log-rank (Mantel-Cox) test. For the three pairwise comparisons of the high-, mid-, and low-dose AAV-treated groups versus untreated *Aldh5a1*^−/−^ mice, a Bonferroni correction was applied, resulting in a corrected significance threshold of *p* < 0.0167. Asterisks indicate pairwise comparisons versus untreated *Aldh5a1*^−/−^ mice. Sample sizes were as follows: untreated *Aldh5a1*^*+/+*^ (*n* = 10), untreated *Aldh5a1*^−/−^ (*n* = 17), saline-treated *Aldh5a1*^−/−^ (*n* = 11), high-dose AAV-treated *Aldh5a1*^−/−^ (*n* = 9), mid-dose AAV-treated *Aldh5a1*^−/−^ (*n* = 6), and low-dose AAV-treated *Aldh5a1*^−/−^ (*n* = 5). ∗∗∗*p* < 0.001; ns, not significant. (B) Longitudinal body weight measurement. Untreated *Aldh5a1*^−/−^ mice demonstrated impaired postnatal weight gain compared to that of *Aldh5a1*^*+/+*^ controls, whereas AAV-treated *Aldh5a1*^−/−^ mice exhibited sustained body weight gain over time. Data are presented as mean ± SD over time. The number of animals contributing data at each time point is provided in Table S2. (C) Body weight at 3 weeks of age. High-dose AAV treatment significantly increased body weight compared with untreated *Aldh5a1*^−/−^ mice and restored body weight to levels comparable with *Aldh5a1*^*+/+*^ controls. Each dot represents an individual mouse and horizontal lines indicate the mean. Sample sizes were untreated *Aldh5a1*^*+/+*^ (*n* = 10), untreated *Aldh5a1*^−/−^ (*n* = 10), saline-treated *Aldh5a1*^−/−^ (*n* = 7), high-dose (*n* = 9), mid-dose (*n* = 5), and low-dose (*n* = 5). Statistical significance was determined by one-way ANOVA with Tukey’s multiple-comparison test. ∗∗*p* < 0.01, ∗∗∗∗*p* < 0.0001; ns, not significant. (D) Motor performance assessed using the rotarod test. Untreated and saline-treated *Aldh5a1*^−/−^ mice exhibited severely impaired motor performance, whereas AAV-treated mice demonstrated sustained enhancement in latency to fall over the observation period. Data are presented as mean ± SD over time. The number of animals contributing data at each time point is provided in Table S3. (E) Rotarod performance at 3 weeks of age. High- and mid-dose AAV vector treatment significantly enhanced motor coordination compared with untreated *Aldh5a1*^−/−^ mice. Low-dose-treated mice showed numerically higher rotarod performance than untreated *Aldh5a1*^−/−^ mice, although this group was not included in formal statistical comparisons because of the limited sample size (*n* = 2). Some mice that were alive and available for body weight measurement were unable to complete the full rotarod testing protocol, consisting of three trials per day over two consecutive days; therefore, the sample sizes for rotarod assessment were smaller than those for body weight in some groups. Dots represent individual mice, and horizontal lines indicate the mean. Sample sizes were untreated *Aldh5a1*^*+/+*^ (*n* = 10), untreated *Aldh5a1*^−/−^ (*n* = 11), saline-treated *Aldh5a1*^−/−^ (*n* = 4), high-dose (*n* = 9), mid-dose (*n* = 4), and low-dose (*n* = 2). Statistical comparisons among the groups other than the low-dose group were performed using one-way ANOVA with Tukey’s multiple-comparison test. ∗*p* < 0.05, and ∗∗∗∗*p* < 0.0001. (F) Representative photograph comparing untreated and high-dose AAV-treated *Aldh5a1*^−/−^ mice at 5 weeks of age, illustrating enhanced growth following AAV vector treatment.

### Alterations in body weight

Untreated and saline-treated *Aldh5a1*^−/−^ mice exhibited impaired postnatal weight gain compared to that of untreated *Aldh5a1*^*+/+*^ mice (Figure 2B). In contrast, AAV-treated *Aldh5a1*^−/−^ mice exhibited sustained body weight gain, throughout the observation period. Measurements at later time points represent surviving animals for which data were available and longitudinal follow-up was ongoing.

At 3 weeks of age, body weight was significantly lower in untreated and saline-treated *Aldh5a1*^−/−^ mice than that in untreated *Aldh5a1*^*+/+*^ mice (Figure 2C, ∗∗*p* < 0.01). High-dose AAV-treated *Aldh5a1*^−/−^ mice exhibited significantly higher body weight than that of untreated *Aldh5a1*^−/−^ mice (∗∗∗∗*p* < 0.0001) and restored it to levels comparable to untreated *Aldh5a1*^*+/+*^ mice (ns). In contrast, neither mid- nor low-dose AAV vector treatment resulted in a statistically significant difference from that of untreated *Aldh5a1*^−/−^ mice (ns).

### Enhancement of motor function

In the rotarod test, untreated and saline-treated *Aldh5a1*^−/−^ mice exhibited impaired motor performance compared with that of *Aldh5a1*^*+/+*^ controls throughout the observation period (Figure 2D). In contrast, AAV-treated *Aldh5a1*^−/−^ mice exhibited sustained enhancement in motor performance over time, indicating long-term preservation of motor coordination. Measurements at later time points represent surviving animals for which data were available and longitudinal follow-up was ongoing. At 3 weeks of age, untreated and saline-treated *Aldh5a1*^−/−^ mice exhibited a significantly reduced latency to fall than that of untreated *Aldh5a1*^*+/+*^ mice (Figure 2E, ∗∗∗∗*p* < 0.0001). High- and mid-dose AAV-treated *Aldh5a1*^−/−^ mice exhibited significantly improved motor coordination compared with untreated *Aldh5a1*^−/−^ mice (∗*p* < 0.05). In contrast, low-dose treatment showed a trend toward improvement, although formal statistical comparison was not performed because of the limited sample size (*n* = 2). Consistent with these quantitative findings, high-dose AAV-treated *Aldh5a1*^−/−^ mice exhibited improved overall appearance and body condition compared with untreated *Aldh5a1*^−/−^ mice (Figure 2F).

### Detection of vector genomes in the CNS and liver

Quantitative polymerase chain reaction (qPCR) analysis demonstrated that vector genomes were widely distributed throughout the CNS, including the cerebrum, cerebellum, and brainstem, with the highest CNS copy number observed in the cerebrum (Figure 3). Notably, vector genome copies were detected at higher levels in the liver than in CNS regions following neonatal administration.^19^^,^^20^

**Figure 3:**
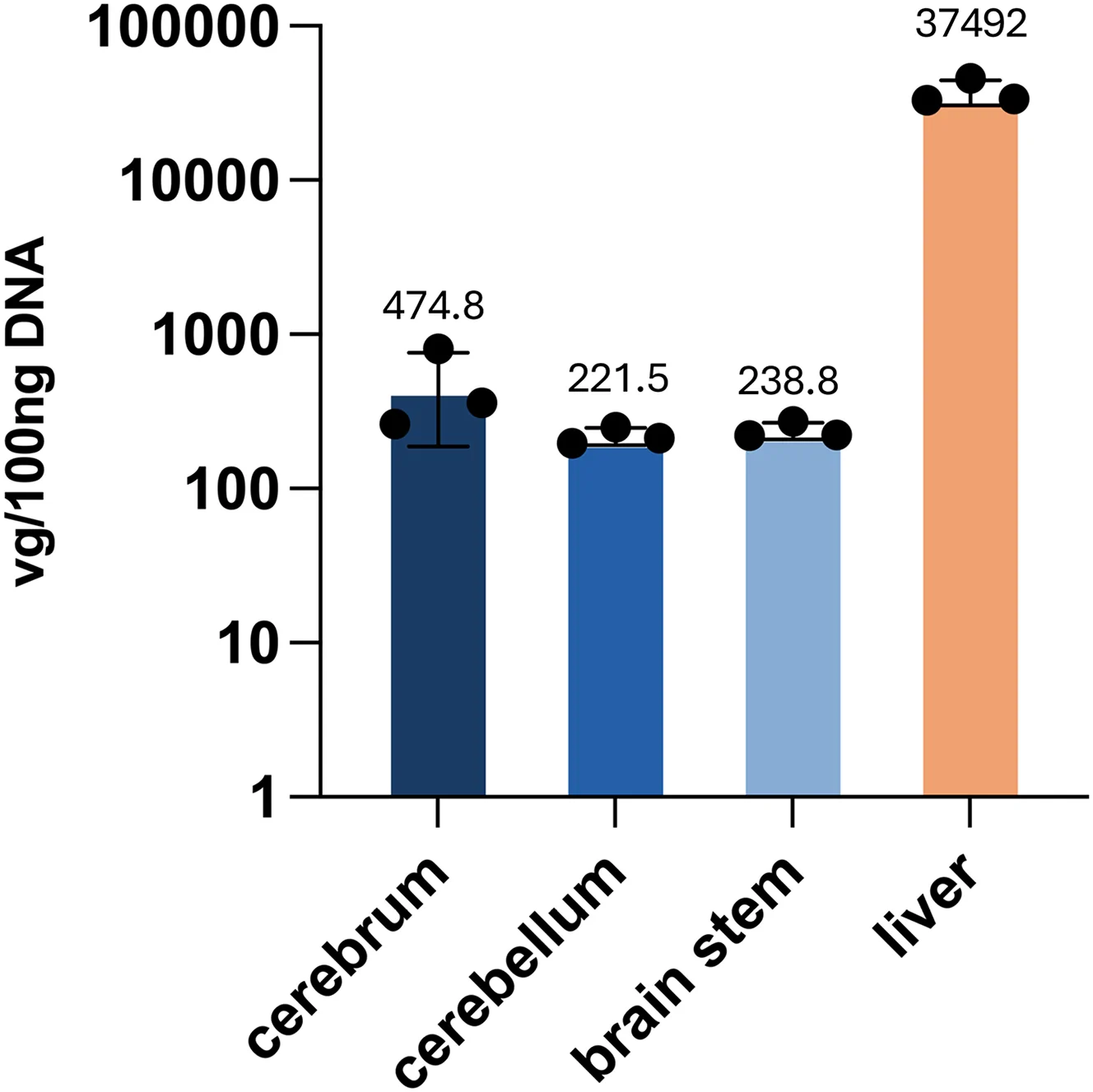
Tissue distribution of adeno-associated virus vector genomes after neonatal sequential intracerebroventricular and cisterna magna administration Vector genome (vg) copy numbers were quantified in the cerebrum, cerebellum, brainstem, and liver using quantitative polymerase chain reaction. Vector genomes were widely detected across brain regions and in the liver. Genomic DNA was extracted from 3-weeks-old mid-dose adeno-associated virus (AAV)-treated aldehyde dehydrogenase 5 family member A1 (*Aldh5a1*) ^−/−^ mice (*n* = 3). Data are expressed as vector genome copies per 100 ng of genomic DNA (vg/100 ng DNA) and presented as the mean ± standard deviation (SD). Each dot represents an individual mouse.

### ALDH5A1 expression following AAV-h*ALDH5A1* vector administration to *Aldh5a1*^−/−^ mice

Western blot analysis demonstrated that, in AAV vector-treated mice, strong bands corresponding to ALDH5A1 (approximately 54 kDa) and Myc-tagged ALDH5A1 (approximately 55.2 kDa) were detected (Figures 4A and S1), indicating robust transgene expression. Endogenous ALDH5A1 was detected in untreated *Aldh5a1*^*+/+*^ mice but was absent in untreated *Aldh5a1*^−/−^ mice. Densitometric analysis revealed ALDH5A1/β-actin ratios of 1.51, 0.002, and 2.36 in untreated *Aldh5a1*^*+/+*^, untreated *Aldh5a1*^−/−^, and low-dose AAV-treated mice, respectively. ALDH5A1 expression was significantly increased in low-dose AAV-treated mice compared with untreated *Aldh5a1*^−/−^ mice (Figure 4B, ∗∗∗∗*p* < 0.0001). Myc expression was also significantly higher in low-dose AAV-treated mice than in untreated *Aldh5a1*^−/−^ mice (Figure 4B, ∗∗∗∗*p* < 0.0001), confirming vector-derived transgene expression. Consistent with these findings, Myc immunofluorescence was detected in the brains of AAV-treated mice, whereas only background-level staining was observed in untreated mice (Figure S2). Myc immunoreactivity showed a heterogeneous and regionally variable distribution in the mid-dose section, whereas a more widespread distribution was observed in the high-dose section.

**Figure 4:**
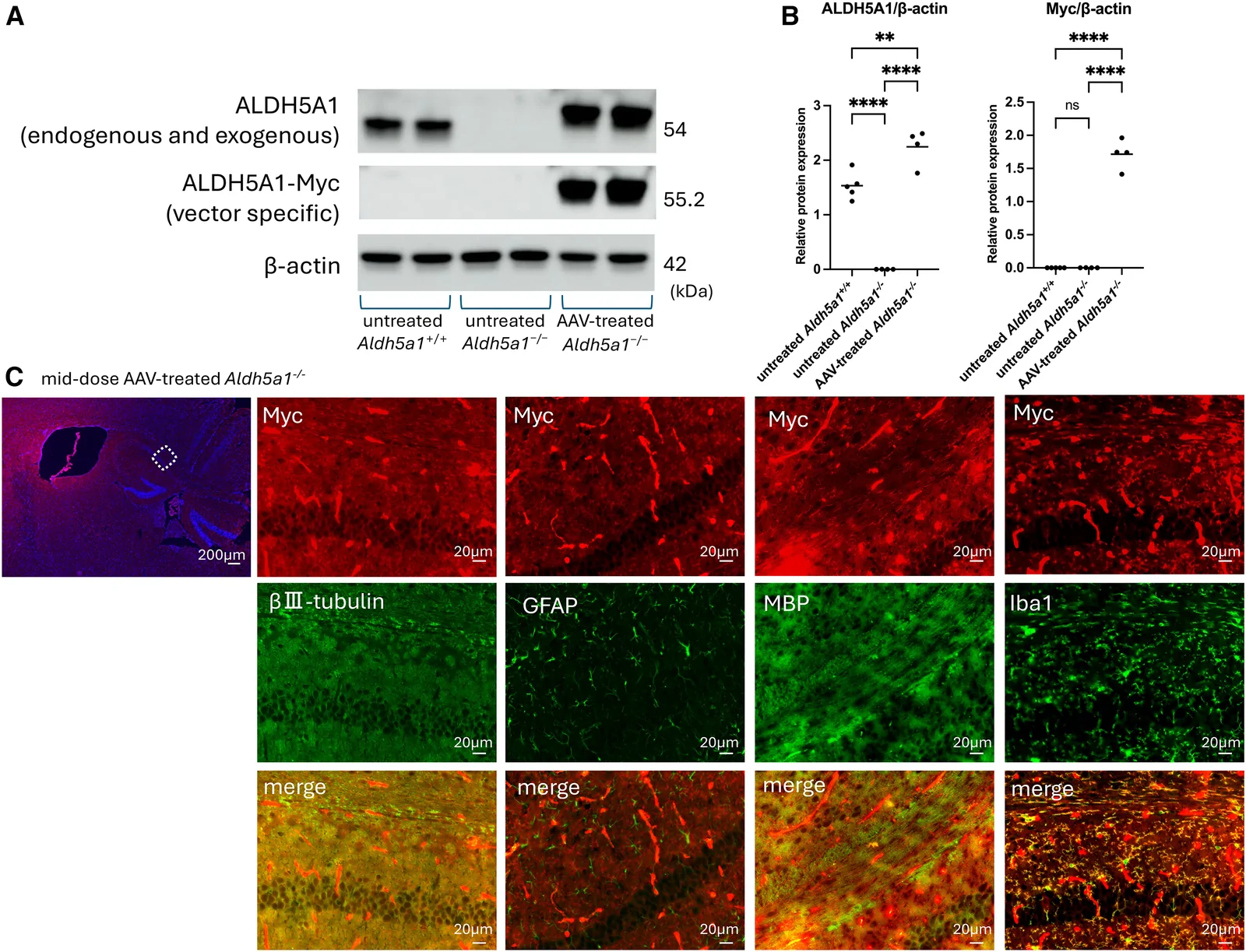
Expression of aldehyde dehydrogenase 5 family member A1 and Myc tag following adeno-associated virus vector treatment (A) Representative western blot analysis of aldehyde dehydrogenase 5 family member A1 (ALDH5A1) protein expression in brain tissue from untreated *Aldh5a1*^*+/+*^ (*n* = 5), untreated *Aldh5a1*^−/−^ (*n* = 4), and low-dose adeno-associated virus (AAV)-treated *Aldh5a1*^−/−^ mice (*n* = 4). Total ALDH5A1 (detecting endogenous and exogenous proteins) and vector-specific ALDH5A1-myc are assessed. β-Actin is used as a loading control. Total ALDH5A1 expression is absent in untreated *Aldh5a1*^−/−^ mice and increased in AAV-treated *Aldh5a1*^−/−^ mice compared to untreated *Aldh5a1*^*+/+*^ mice. (B) Quantification of ALDH5A1 and Myc protein levels normalized to β-actin. Data are presented as relative protein expression levels. Total ALDH5A1 levels were significantly higher in low-dose AAV-treated mice than in untreated *Aldh5a1*^*+/+*^ and untreated *Aldh5a1*^−/−^ mice. Each dot represents an individual mouse, and horizontal lines indicate the mean. Statistical significance was determined using one-way analysis of variance followed by Tukey’s multiple comparison test. ∗∗*p* < 0.01; ∗∗∗∗*p* < 0.0001; ns, not significant. (C) Immunofluorescence analysis of Myc-tagged ALDH5A1 expression in the hippocampus of a mid-dose AAV-treated *Aldh5a1*^−/−^ mouse (*n* = 1). The low-magnification Myc/DAPI merged image of the left cerebral hemisphere indicates the approximate hippocampal region used for high-magnification imaging (dashed outline) and was reused in Figure S2 to demonstrate the distribution of Myc immunoreactivity. Myc (red) was co-stained with βIII-tubulin (neurons), glial fibrillary acidic protein (GFAP; astrocytes), myelin basic protein (MBP; myelin), or ionized calcium-binding adapter protein 1 (Iba1; microglia) (green). Merged high-magnification images show apparent overlap of Myc immunoreactivity with βIII-tubulin, MBP, and Iba1 signals. Scale bars: 200 μm (low-magnification image) and 20 μm (high-magnification images).

### Myc-tagged SSADH expression and overlap with cell-type markers in the brains of AAV vector-treated mice

Immunofluorescence staining revealed apparent overlap of Myc-tagged exogenous SSADH immunoreactivity with βIII-tubulin, myelin basic protein (MBP), and ionized calcium-binding adapter protein 1 (Iba1) signals in mid-dose AAV-treated *Aldh5a1*^−/−^ mice (Figure 4C). In contrast, apparent overlap with glial fibrillary acidic protein (GFAP) immunoreactivity was not observed.

### Qualitative histological changes following AAV vector treatment

Histological examination revealed qualitative differences in ventricular and hippocampal morphology among representative sections from untreated *Aldh5a1*^+/+^, untreated *Aldh5a1*^−/−^, and mid-dose AAV-treated *Aldh5a1*^−/−^ mice (Figure S3). Ventricular enlargement and altered hippocampal morphology were observed in untreated *Aldh5a1*^−/−^ mice, whereas these abnormalities appeared less pronounced in the representative mid-dose AAV-treated section.

### Astrocyte activation assessed by GFAP immunoreactivity

Astrogliosis was assessed by GFAP immunostaining. Untreated *Aldh5a1*^−/−^ mice exhibited increased GFAP immunoreactivity in the hippocampus, consistent with astrogliosis (Figure 5A). Similar findings were observed in other brain regions, although only representative hippocampal images are shown. In contrast, GFAP immunoreactivity tended to be reduced in mid-dose AAV-treated mice.

**Figure 5:**
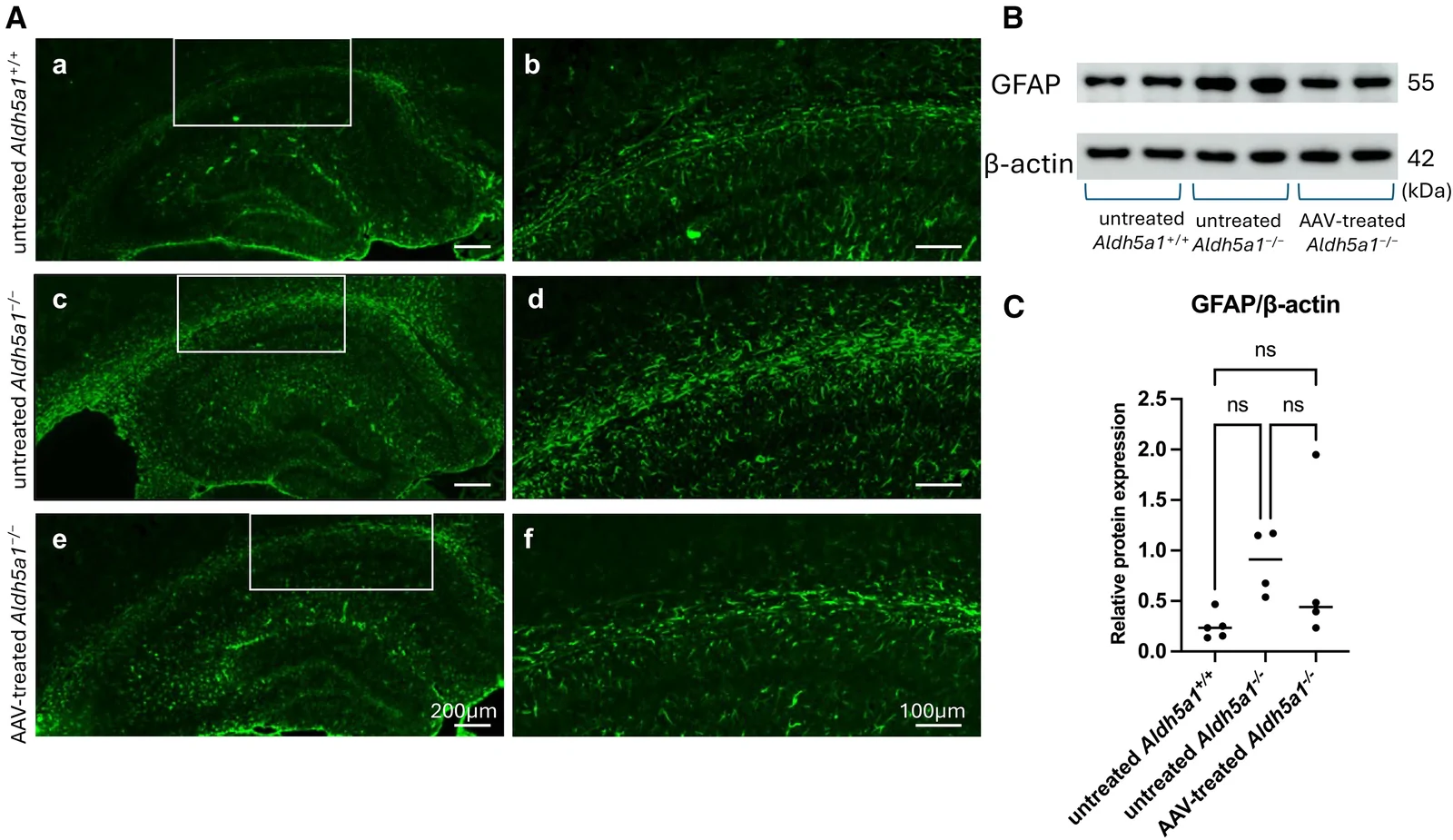
Astrocytic alterations following adeno-associated virus vector treatment (A) Representative immunofluorescence images of glial fibrillary acidic protein (GFAP) staining in hippocampal sections from untreated aldehyde dehydrogenase 5 family member A1 (*Aldh5a1*)^*+/+*^, untreated *Aldh5a1*^−/−^, and mid-dose adeno-associated virus (AAV)-treated *Aldh5a1*^−/−^ mice. The biological sample sizes for this imaging analysis were untreated *Aldh5a1*^*+/+*^ (*n* = 2), untreated *Aldh5a1*^−/−^ (*n* = 2), and mid-dose AAV-treated *Aldh5a1*^−/−^ (*n* = 1). Untreated *Aldh5a1*^−/−^ mice exhibited qualitatively increased GFAP immunoreactivity compared with untreated *Aldh5a1*^*+/+*^ mice, whereas AAV-treated *Aldh5a1*^−/−^ mice showed an apparent reduction in GFAP staining. (B, D, and F) correspond to the boxed regions in (A, C, and E), respectively. The same tissue sections were independently imaged at higher magnification for the GFAP/Iba1 analysis shown in Figure 6. Scale bars: 200 μm (low magnification) and 100 μm (high magnification). (B) Representative western blot analysis of GFAP protein expression in right-hemisphere brain lysates from untreated *Aldh5a1*^*+/+*^ (*n* = 5), untreated *Aldh5a1*^−/−^ (*n* = 4), and mid-dose AAV-treated *Aldh5a1*^−/−^ mice (*n* = 4). β-Actin is used as a loading control. (C) Quantification of GFAP protein levels normalized to β-actin. Data are presented as relative expression levels. GFAP expression was increased in untreated *Aldh5a1*^−/−^ mice compared with untreated *Aldh5a1*^*+/+*^ mice. Although GFAP levels appeared lower in AAV-treated *Aldh5a1*^−/−^ mice, no significant difference was observed between untreated and AAV-treated *Aldh5a1*^−/−^ mice. Each dot represents an individual mouse, and horizontal lines indicate the mean. Statistical significance was determined using one-way analysis of variance followed by Tukey’s multiple comparison test. ns, not significant.

Consistently, western blot analysis revealed an increased GFAP/β-actin ratio in untreated *Aldh5a1*^−/−^ mice, whereas mid-dose AAV-treated mice reduced the ratio toward levels comparable to untreated *Aldh5a1*^*+/+*^ mice (Figure 5B). Although quantitative analysis demonstrated significant inter-individual variability and did not reach statistical significance among groups, AAV-treated mice exhibited a trend toward reduced reactive astrogliosis (Figure 5C).

### Assessment of astrocyte and microglial activation

To qualitatively assess neuroinflammatory changes, double immunofluorescence staining for GFAP and Iba1 was performed in hippocampal sections. Untreated *Aldh5a1*^−/−^ mice exhibited increased GFAP and Iba1 immunoreactivity compared with untreated *Aldh5a1*^*+/+*^ mice, consistent with astrocyte and microglial activation (Figure 6). In contrast, GFAP and Iba1 immunoreactivity appeared reduced in mid-dose AAV-treated mice. GFAP-positive astrocytes and Iba1-positive microglia were observed within the same hippocampal regions; however, the present imaging was not designed to assess direct astrocyte-microglia interactions.

**Figure 6:**
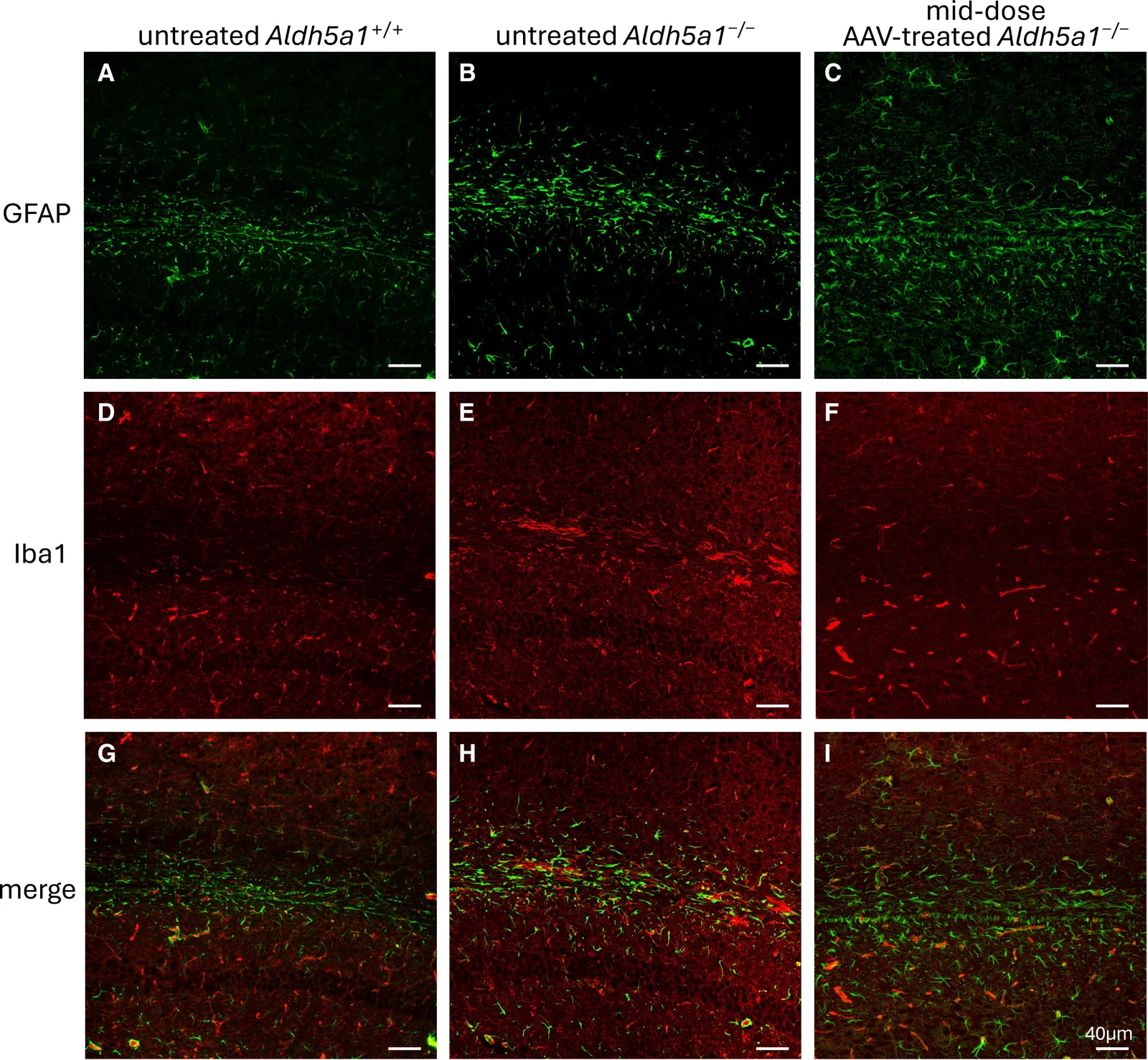
Effects of adeno-associated virus vector-mediated gene replacement on astrocyte and microglial activation in the aldehyde dehydrogenase 5 family member A1^−/−^ mouse brain Immunofluorescence analysis of glial fibrillary acidic protein (GFAP) (green) and ionized calcium-binding adapter protein 1 (Iba1) (red) expression in hippocampal sections from untreated aldehyde dehydrogenase 5 family member A1 (*Aldh5a1*)^*+/+*^ (*n* = 1), untreated *Aldh5a1*^−/−^ (*n* = 1) and mid-dose adeno-associated virus (AAV)-treated *Aldh5a1*^−/−^ mice (*n* = 1). Panels (A–C), (D–F), and (G–I) show GFAP, Iba1, and merged images, respectively. In each row, the left, middle, and right panels represent untreated *Aldh5a1*^*+/+*^, untreated *Aldh5a1*^*−/−*^, and mid-dose AAV-treated *Aldh5a1*^*−/−*^ mice, respectively. Both GFAP and Iba1 immunoreactivity appeared increased in untreated *Aldh5a1*^−/−^ mice compared with untreated *Aldh5a1*^*+/+*^ mice. GFAP and Iba1 immunoreactivity appeared reduced in mid-dose AAV-treated *Aldh5a1*^−/−^ mice compared with untreated *Aldh5a1*^−/−^ mice. Merged images show GFAP-positive astrocytes and Iba1-positive microglia within the same hippocampal regions. The present imaging was not intended to assess direct astrocyte-microglia interactions. The images were independently acquired at higher magnification from the same tissue sections shown in Figure 5A. Scale bars, 40 μm.

### Restoration of myelination following AAV vector treatment

Myelination in the hippocampus was assessed using MBP immunostaining. Untreated *Aldh5a1*^−/−^ mice exhibited reduced MBP immunoreactivity in the hippocampus compared with untreated *Aldh5a1*^*+/+*^ mice, consistent with impaired myelination (Figure 7A). In contrast, MBP immunoreactivity appeared partially preserved in mid-dose AAV-treated mice, particularly along hippocampal fiber tracts.

**Figure 7:**
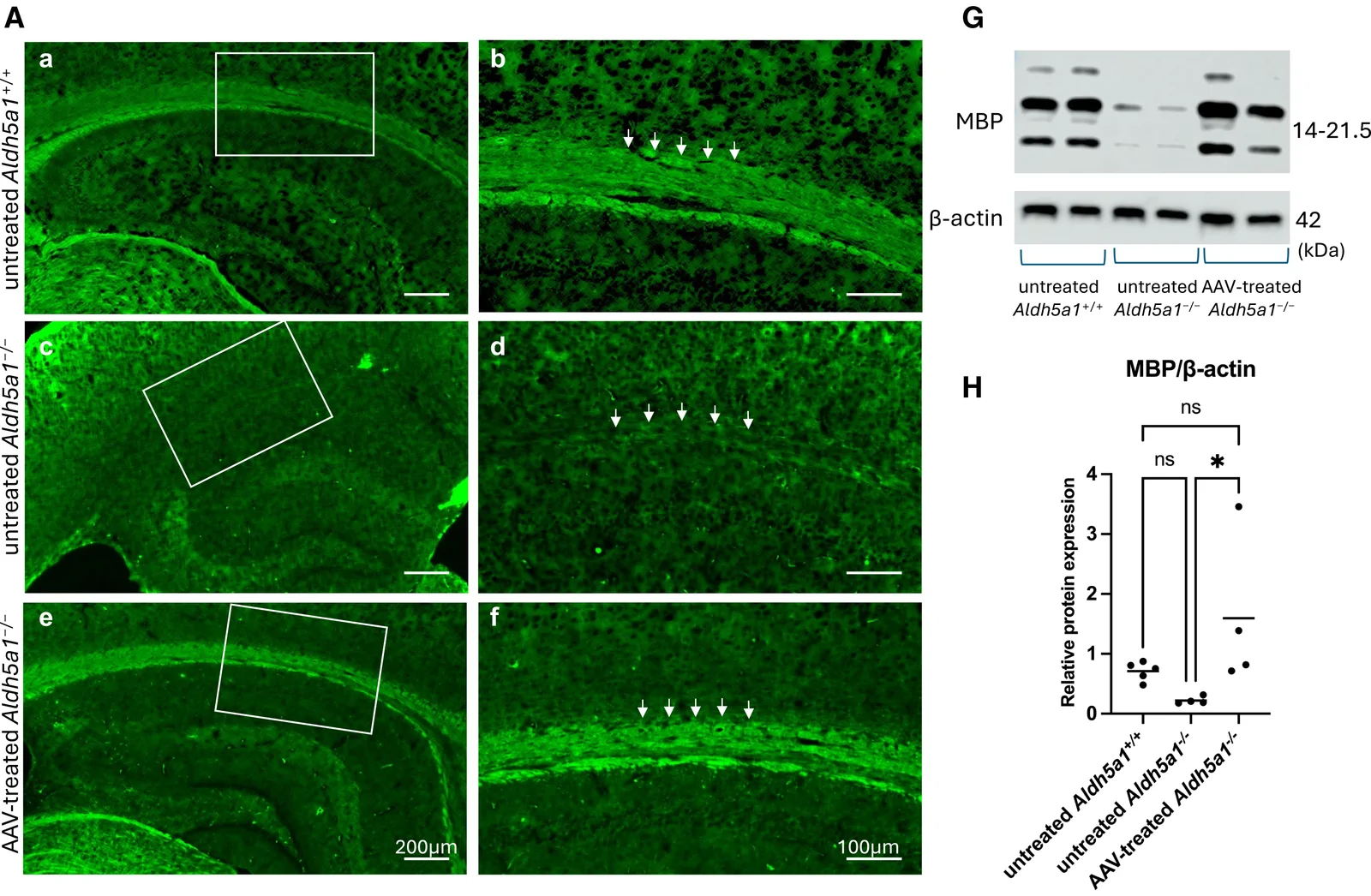
Myelin basic protein expression following adeno-associated virus vector treatment (A) Representative immunofluorescence images of myelin basic protein (MBP) staining in hippocampal sections from untreated aldehyde dehydrogenase 5 family member A1 (*Aldh5a1*)^*+/+*^, untreated *Aldh5a1*^−/−^, and mid-dose adeno-associated virus (AAV)-treated *Aldh5a1*^−/−^ mice. The biological sample sizes for this imaging analysis were untreated *Aldh5a1*^*+/+*^ (*n* = 2), untreated *Aldh5a1*^−/−^ (*n* = 2), and mid-dose AAV-treated *Aldh5a1*^−/−^ (*n* = 1). Untreated *Aldh5a1*^−/−^ mice exhibited qualitatively reduced MBP immunoreactivity in the hippocampal region compared with untreated *Aldh5a1*^*+/+*^ mice, whereas MBP staining appeared increased in AAV-treated *Aldh5a1*^−/−^ mice. Higher-magnification images (b, d, and f) correspond to the regions indicated in (a, c, and e), respectively. Arrows indicate myelinated fiber layers. Scale bars: 200 μm (low magnification) and 100 μm (high magnification). (G) Representative western blot analysis of MBP protein expression in right hemisphere brain lysates from untreated *Aldh5a1*^*+/+*^ (*n* = 5), untreated *Aldh5a1*^−/−^ (*n* = 4), and mid-dose AAV-treated *Aldh5a1*^−/−^ mice (*n* = 4). MBP quantification represents the summed intensity of all immunoreactive MBP bands normalized to β-actin, which was used as a loading control. Full western blot images are provided in Figure S4. (H) Quantification of MBP protein levels normalized to β-actin. Data are presented as relative expression levels. MBP expression was significantly higher in mid-dose AAV-treated *Aldh5a1*^−/−^ mice than in untreated *Aldh5a1*^−/−^ mice. Although MBP levels showed considerable inter-individual variability in AAV-treated mice, the difference between AAV-treated *Aldh5a1*^−/−^ and untreated *Aldh5a1*^*+/+*^ mice was not statistically significant. Each dot represents an individual mouse, and horizontal lines indicate the mean. Statistical significance was determined using one-way analysis of variance followed by Tukey’s multiple comparison test. ∗*p* < 0.05; ns, not significant.

Consistent with these histological findings, western blot analysis demonstrated reduced MBP protein expression in untreated *Aldh5a1*^−/−^ mice compared with untreated *Aldh5a1*^*+/+*^ controls, whereas MBP expression was increased in mid-dose AAV-treated mice (Figure 7G; full blots shown in Figure S4). Quantification of MBP signal intensity normalized to β-actin showed significantly higher MBP expression in mid-dose AAV-treated *Aldh5a1*^−/−^ mice than in untreated *Aldh5a1*^−/−^ mice (*p* < 0.05; Figure 7H). Although MBP levels in some AAV-treated mice exceeded those observed in untreated *Aldh5a1*^*+/+*^ mice, considerable inter-individual variability was observed, and the difference between these groups was not statistically significant. These findings suggest partial preservation of myelination following AAV vector treatment.

### Alterations in urinary GABA and GHB levels

Urinary GABA and GHB concentrations, normalized to urinary creatinine and expressed as mmol/mol creatinine, were significantly elevated in untreated *Aldh5a1*^−/−^ mice during the early postnatal period compared with *Aldh5a1*^*+/+*^ controls (Figure 8). Following AAV vector administration, a rapid biochemical response was observed. At 1 week post-treatment, high-dose AAV treatment was associated with marked reductions in both urinary GABA and GHB levels. Notably, urinary GHB concentrations were reduced to near-baseline levels and were undetectable in several high-dose-treated mice. At 1 week post-treatment, both high- and low-dose AAV-treated mice showed significantly lower urinary GABA and GHB concentrations than untreated *Aldh5a1*^−/−^ mice (one-way ANOVA with Tukey’s multiple comparison test, *p* < 0.0001 for all comparisons), whereas no significant difference was observed between the high- and low-dose groups. Interpretation of the mid-dose group was limited by the small sample size.

**Figure 8:**
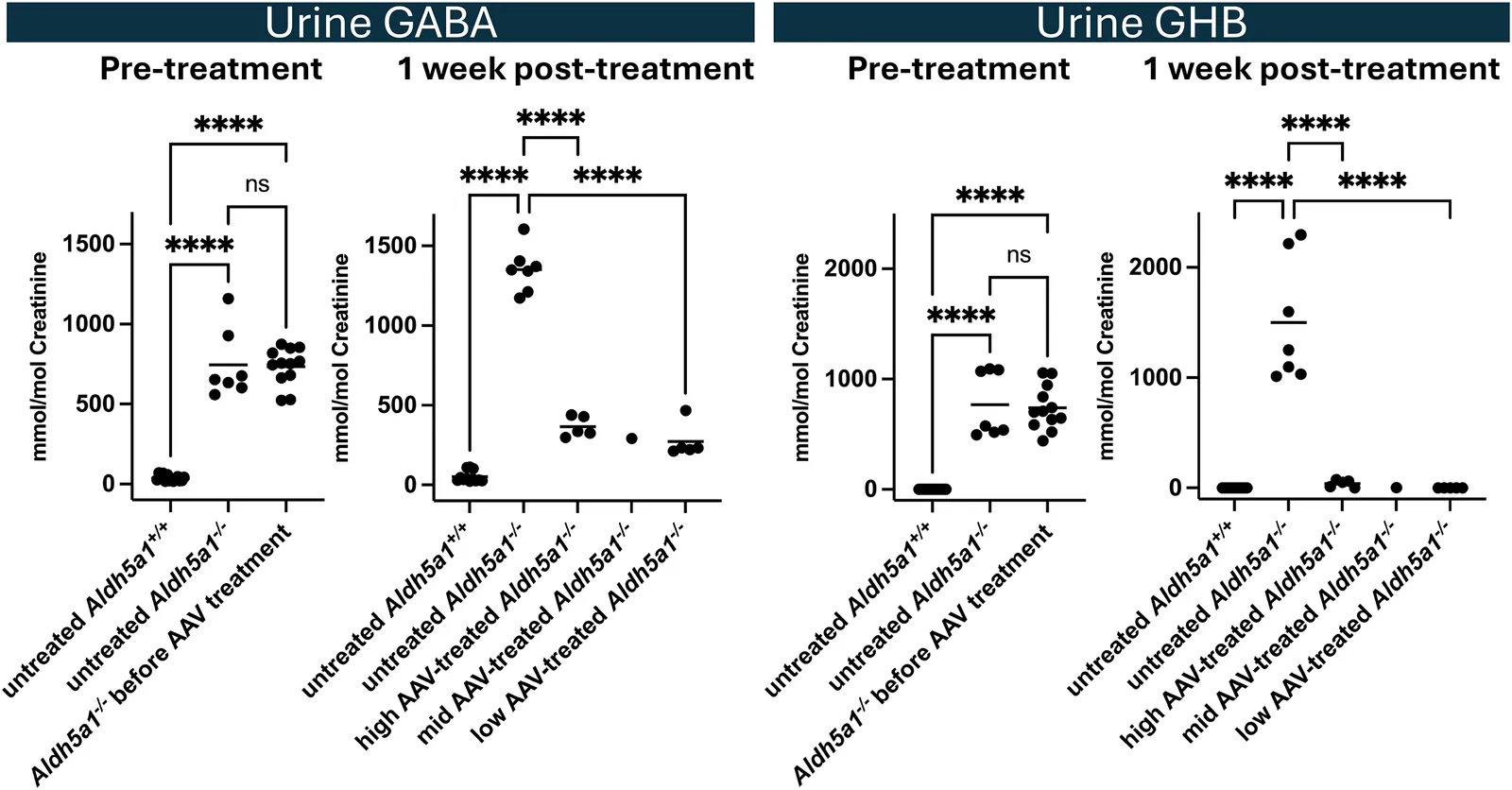
Changes in urinary γ-aminobutyric acid and γ-hydroxybutyrate levels following adeno-associated virus vector treatment Pre-treatment samples obtained before adeno-associated virus (AAV) vector administration demonstrated significantly increased urinary γ-aminobutyric acid (GABA) and γ-hydroxybutyrate (GHB) levels in aldehyde dehydrogenase 5 family member A1 (*Aldh5a1*)^−/−^ mice compared with *Aldh5a1*^*+/+*^ controls. In untreated *Aldh5a1*^−/−^ mice, both metabolites further increased by 1 week, indicating progressive postnatal accumulation. In contrast, high- and low-dose AAV-treated mice exhibited lower urinary GABA and GHB levels at 1 week post-administration. Urinary GABA and GHB concentrations were measured by liquid chromatography-tandem mass spectrometry (LC-MS/MS), normalized to urinary creatinine concentrations, and expressed as mmol/mol creatinine. Group comparisons at each time point were performed using one-way ANOVA followed by Tukey’s multiple comparison test. ∗∗∗∗*p* < 0.0001; ns, not significant. Sample sizes were as follows: pre-treatment, untreated *Aldh5a1*^*+/+*^ (*n* = 14), untreated *Aldh5a1*^−/−^ (*n* = 7), and *Aldh5a1*^−/−^ before AAV treatment (*n* = 12); 1 week, *Aldh5a1*^*+/+*^ (*n* = 14), untreated *Aldh5a1*^−/−^ (*n* = 7), high-dose (*n* = 5), mid-dose (*n* = 1), and low-dose (*n* = 5). Interpretation of the mid-dose group is limited because only a single sample was available at this time point. Pre-treatment samples were obtained from 12 mice subsequently assigned to AAV treatment; however, urine could not be collected from one mouse at the 1-week time point, resulting in 11 evaluable samples. Animals included in the urinary metabolite analysis partially overlapped with the longitudinal cohort described in the materials and methods, with additional animals included specifically for metabolite analysis.

## Discussion

This study demonstrated that AAV vector-mediated *ALDH5A1* gene replacement confers robust survival benefit and exploratory histological findings consistent with partial attenuation of neuroglial pathology in a severe mouse model of SSADH deficiency, supporting further development of CNS-directed AAV vector gene therapy in SSADH deficiency. AAV vector treatment resulted in sustained extension of survival, recovery of body weight, long-term enhancement in motor performance, and significant reduction of GABA and GHB levels in urine. Together, these findings support *ALDH5A1* gene replacement as a promising therapeutic strategy for SSADH deficiency.

The SSADH-deficient mouse model exhibits a severe phenotype characterized by early-onset seizures, ataxia, and premature death, typically within the first month of life.^14^^,^^15^^,^^16^^,^^17^ Previous therapeutic approaches, including pharmacological modulation of GABAergic signaling,^14^^,^^15^^,^^21^ metabolic supplementation,^9^^,^^22^ enzyme replacement,^23^ and adenoviral gene transfer,^13^ have provided only transient or modest benefits without durable metabolic correction or long-term survival enhancement. These limitations likely reflected insufficient correction of CNS pathology. In particular, liver-directed gene transfer achieved substantial peripheral expression but only partial phenotypic improvement, suggesting that correction of central metabolic abnormalities is required for meaningful therapeutic benefit. In contrast, this study demonstrated restoration of SSADH expression within the CNS, which may have contributed to the metabolic and therapeutic improvements observed during the study period.

SSADH deficiency leads to prenatal and postnatal accumulation of GABA and GHB, disrupting excitatory-inhibitory balance and impairing neuronal circuit formation, with associated glial activation and myelination deficits.^8^^,^^9^^,^^10^^,^^11^ Recent studies have further demonstrated central dysmyelination in both individuals with SSADH deficiency and SSADH-deficient mice. Reduced expression of multiple myelin-related genes, including MBP, MOBP, MAL, ASPA, and SLC25A13, has been reported in the hippocampus of SSADH-deficient mice, suggesting that chronic metabolic abnormalities may impair oligodendrocyte maturation and myelin maintenance.^24^ Chronic metabolic disturbance promotes network hyperexcitability and neuroglial pathology. Our findings extend these observations by demonstrating partial restoration of MBP expression and exploratory histological findings consistent with attenuation of astrogliosis following *ALDH5A1* gene replacement therapy, suggesting that correction of the underlying metabolic defect may ameliorate both dysmyelination and neuroglial pathology in SSADH deficiency.

Although some biochemical and behavioral improvements were observed at lower doses, the survival benefit was greater and more consistent at higher doses. These findings suggest the presence of an apparent therapeutic threshold for robust survival rescue, although the basis for the dose-dependent differences in survival remains uncertain.

Vector genomes were detected throughout the CNS and in the liver, indicating that neonatal administration facilitated both systemic and central vector delivery. Although hepatic vector genome levels exceeded those detected in the CNS, vector genomes were widely distributed throughout the CNS. These findings raise the possibility that therapeutic efficacy may reflect contributions from both CNS and peripheral transduction; however, their relative contributions cannot be determined from the present study because metabolic correction was not assessed separately in CNS and peripheral tissues.

Compared with other CNS gene therapy models, the AAV vector dose required for survival rescue in SSADH deficiency was relatively high.^19^^,^^25^ ALDH5A1 expression levels in treated mice exceeded those observed in wild-type controls, even in the low-dose group. However, despite supraphysiological ALDH5A1 expression, the low-dose group showed no survival benefit. Because western blot analysis measures total protein abundance in whole-brain lysates rather than the regional distribution or cellular extent of transgene expression, these findings do not necessarily indicate adequate correction throughout the CNS. Low-magnification Myc immunofluorescence demonstrated heterogeneous and regionally variable transgene distribution in the mid-dose section and a more widespread distribution in the high-dose section. However, spatial transgene distribution was not evaluated in the low-dose group. Therefore, whether differences in CNS transgene distribution contributed to the lack of therapeutic benefit in the low-dose group cannot be determined from the present study. Although supraphysiological or ectopic ALDH5A1 expression could theoretically have unintended effects, our data provide no direct evidence that off-target expression accounted for the lack of survival benefit observed in the low-dose group.

Despite apparent overlap with neuronal, oligodendroglial, and microglial markers, astrocytic expression was not detected under the conditions examined. This may reflect vector tropism, cell type-specific promoter activity, or limited sensitivity of immunofluorescence detection.

From a translational perspective, the apparent therapeutic threshold observed in this study may inform dose selection for future clinical development. Future studies should evaluate clinically feasible delivery approaches, such as intrathecal administration, and determine whether comparable efficacy can be achieved after symptom onset.

Several limitations should be acknowledged. Seizure monitoring and electroencephalogram (EEG) analyses were not performed; therefore, treatment effects on epileptic activity could not be directly assessed. Histological analyses were exploratory and not designed to evaluate dose-response relationships, and some neuroinflammatory findings were based on qualitative immunohistochemical assessment. In addition, because of the limited sample size, considerable inter-individual variability was observed in some histological and protein expression analyses, including GFAP and MBP. Therefore, these findings should be interpreted cautiously and require confirmation in larger cohorts. Abrupt metabolic correction in a chronically hyper-GABAergic brain may transiently disrupt inhibitory signaling.^2^ Furthermore, neonatal intracerebroventricular (i.c.v.) and cisterna magna administration may not fully reflect clinically feasible treatment paradigms, and therapeutic efficacy is likely influenced by developmental timing. Finally, vector doses required for efficacy in mice may not directly translate to humans, and long-term safety—including immune responses, durability of transgene expression, and cytomegalovirus (CMV) promoter performance—remains to be established.

In summary, AAV vector-mediated *ALDH5A1* gene therapy improved metabolic abnormalities, demonstrated exploratory histological findings consistent with partial attenuation of neuroglial pathology, and prolonged survival in a severe mouse model of SSADH deficiency. These findings support further development of *ALDH5A1* gene replacement therapy for SSADH deficiency and suggest an apparent therapeutic threshold for survival benefit. Together, these results provide preclinical support for clinical translation while highlighting the need for careful optimization of dosing strategies and long-term safety assessment. The therapeutic benefit observed following early treatment further highlights the potential importance of early diagnosis and intervention before irreversible neurological injury develops.

## Materials and methods

### Production of recombinant AAV vectors

The AAV vector plasmid contained an expression cassette comprising the CMV immediate-early promoter, a human growth hormone (hGH) intron, human *ALDH5A1* cDNA (GenBank: NM_001080), C-terminal Myc and DYKDDDK(DDK) epitope tags, the woodchuck hepatitis virus post-transcriptional regulatory element (WPRE), and the simian virus 40 polyadenylation signal sequence, flanked by the inverted terminal repeats of the AAV3 genome (Figure 1B). The total expression cassette was 4,176 bp in length and contained a 1,605-bp h*ALDH5A1* coding sequence. A tyrosine-mutant AAV9 capsid (Y446F and Y731F) that enhances neuronal transduction was used.^26^^,^^27^

Recombinant AAV vectors were produced by the transient transfection of HEK293 cells using the vector plasmid (AAV3 rep), tyrosine-mutant AAV9 vp expression plasmid, and adenoviral helper plasmid pHelper (Agilent Technologies, Santa Clara, CA, USA), as described previously.^28^ The recombinant viruses were purified using two sequential continuous CsCl gradients, and viral titers were determined by qPCR.

### *Aldh5a1* deficient mice and genotyping

We ordered B6.129-*Aldh5a1*^tm1Kmg^/J mice (strain #006257) from The Jackson Laboratory (Bar Harbor, ME, USA) and established a breeding colony by crossing heterozygous mice. The mice were housed in individually ventilated cages in a specific pathogen-free, humidity- and temperature-controlled facility (22°C–24°C, 48% relative humidity) under a 12 h light/dark cycle, with free access to water and standard rodent chow (RodentDiet; CLEA Japan, Tokyo, Japan). All experimental procedures involving mice were reviewed and approved by the Institutional Animal Care and Use Committee of Jichi Medical University (approval nos. 20158 and 24093). Experiments involving recombinant AAV vectors were reviewed and approved by the Institutional Biosafety Committee of Jichi Medical University (approval nos. R4-104 and R7-96). All experiments were conducted in accordance with the guidelines of the Ministry of Education, Culture, Sports, Science and Technology (MEXT), Japan.

Genomic DNA was extracted from tail tips collected at postnatal days 2–3 and used for PCR-based genotyping. Wild-type and mutant *Aldh5a1* alleles were identified using allele-specific primer sets and PCR conditions described in Table S1.

### AAV-h*ALDH5A1* vector injection and comparison of the phenotype with controls

The AAV-h*ALDH5A1* vector was administered at three dose levels—high (2.5 × 10^11^ vg), mid (1.5 × 10^11^ vg), and low (0.7 × 10^11^ vg)—to evaluate the impact of vector dose on therapeutic outcomes. The tyrosine-mutant AAV9/3-CMV-h*ALDH5A1* vector was administered at postnatal days 4–5 by sequential administration into the left lateral ventricle (5 μL), followed by the cisterna magna (5 μL) (Figure 1C). Saline-treated control mice received saline using the same injection procedure.

Cooling anesthesia was applied before injection, and mice were recovered on a 37°C hot plate before being returned to their mothers. The survival cohort comprised six groups: untreated *Aldh5a1*^*+/+*^ (*n* = 10), untreated *Aldh5a1*^−/−^ (*n* = 17), saline-treated *Aldh5a1*^−/−^ (*n* = 11), and high- (*n* = 9), mid- (*n* = 6), and low-dose (*n* = 5) AAV-treated *Aldh5a1*^−/−^ groups. Body weight and rotarod performance were assessed longitudinally in animals from these groups for which measurements were available at each scheduled time point. Because of mortality, unavailable measurements, and completion of longitudinal phenotypic follow-up for individual animals, the number of animals contributing to the body weight and rotarod analyses varied across time points. The actual number of animals contributing data at each time point is provided in Tables S2 and S3 for body weight and rotarod performance, respectively. For the low-dose group, body weight measurements were not obtained at weeks 1 and 2. Measurements were available for five mice at week 3 and for one mouse at week 4; because only a single measurement was available at week 4, this time point was not included in graphical group comparisons or statistical analyses.

Formal randomization was not performed. Following genotyping, *Aldh5a1*^−/−^ mice were assigned to the saline-, low-, mid-, or high-dose groups as animals became available, without the use of a predefined randomization scheme. During the initial phase of the study, investigators performing AAV vector injections in this cohort were blinded to the vector dose identity. This applied to 5 of 9 high-dose mice, 5 of 6 mid-dose mice, and 3 of 5 low-dose mice. In the later phase, additional animals were assigned to specific dose groups to supplement group sample sizes; therefore, investigators were aware of the vector dose at the time of administration for the remaining 4 high-dose, 1 mid-dose, and 2 low-dose mice. The vector dose identity for the blinded administrations was disclosed by the investigator who prepared the vectors after administration. Animals used for the urinary metabolite analysis shown in Figure 8 partially overlapped with the survival cohort described earlier and depicted in Figure 1D; additional animals were included specifically for metabolite analysis. Separate cohorts were sacrificed at 3 weeks of age for molecular and histological analyses. Accordingly, the sample sizes for the urinary metabolite, molecular, and histological analyses differ from those of the survival cohort and are provided in the corresponding figure legends.

Both male and female mice were included in longitudinal assessments; however, the study was not designed to evaluate sex-specific treatment effects.

The overall study design and assessment schedule are summarized in Figure 1D.

### Analysis of survival, body weight, and rotarod performance

Survival was assessed as the primary endpoint using Kaplan-Meier analysis. Survival time was defined as the number of days from birth to death. Death from any cause during the observation period was considered a survival event. Mice that remained alive when survival follow-up was terminated were administratively censored on the last day of observation. Body weight and motor function were assessed longitudinally based on the experimental timeline (Figure 1D). Motor coordination was assessed using an accelerating rotarod apparatus (MK-610A; Muromachi Kikai, Tokyo, Japan). The mice were placed on a rotating rod (30 mm diameter) and underwent two consecutive days of training, with three trials per day. Thirty minutes after completion of the third training trial on the second day, a separate test trial was performed, and the latency to fall during this test trial was used as the final value for each mouse. The rotarod accelerated from 4 to 40 rpm over 300 s, and the maximum trial duration was 1,200 s. Rotarod testing was not performed in a blinded manner. However, latency to fall was recorded objectively using an automated rotarod apparatus.

### Brain tissue preparation

At 3 weeks of age, the mice were anesthetized with isoflurane. Subsequently, whole blood was collected by cardiac puncture, followed by euthanasia and rapid brain dissection. Brain tissues were processed for histological, immunohistochemical, and molecular analyses. These analyses were performed using separate cohorts and were intended as exploratory assessments of tissue-level effects rather than dose-response relationships. Group sizes for each analysis are provided in the corresponding figure legends.

### Quantification of AAV-h*ALDH5A1* vector DNA using qPCR

Genomic DNA was extracted from the cerebrum, cerebellum, brainstem, and liver of mid-dose AAV-treated *Aldh5a1*^−/−^ mice at 3 weeks of age using the NucleoSpin TriPrep kit (Macherey-Nagel, Düren, Germany). Vector biodistribution across CNS regions and peripheral tissues was quantified by qPCR using 20 ng of genomic DNA with TaqMan Fast Universal PCR Master Mix (Thermo Fisher Scientific, Waltham, MA, USA) and a human *ALDH5A1*-specific TaqMan assay (Hs00542449_m1; FAM-MGB, Thermo Fisher Scientific) on an Applied Biosystems 7500 Fast Real-Time PCR System (Thermo Fisher Scientific). Absolute quantification was performed using a standard curve generated from serial dilutions (10^1^–10^7^ vg/μL) of vector DNA. This analysis was performed to confirm vector biodistribution and was not designed to evaluate dose-response relationships.

### H&E staining

To assess gross brain morphology and cytoarchitecture, tissues were processed, sectioned, and stained with hematoxylin and eosin (H&E) by Genostaff Co., Ltd. (Tokyo, Japan) using standard histological procedures.

### Immunofluorescence staining

Brains were fixed in 4% paraformaldehyde (FUJIFILM Wako, Osaka, Japan), cryoprotected in sucrose, embedded in optimal cutting temperature compound (Sakura Finetek Japan, Japan), and sectioned coronally at 10 μm thickness.

Sections were blocked with phosphate-buffered saline containing 4% goat serum (Sigma-Aldrich, St. Louis, MO, USA) and 0.1% Triton X-100 (FUJIFILM Wako, Osaka, Japan), and incubated with primary antibodies followed by appropriate fluorescent secondary antibodies (Table S4). Fluorescent images were acquired using a BZ-X800 fluorescence microscope (Keyence, Osaka, Japan).

Immunofluorescence analyses were performed to assess the overlap of exogenous SSADH and associated neuroglial pathology, using the left cerebral hemisphere from untreated *Aldh5a1*^*+/+*^ mice, untreated *Aldh5a1*^−/−^ mice, and mid-dose AAV-treated *Aldh5a1*^−/−^ mice. Unless otherwise specified, immunofluorescence analyses were performed in the hippocampal region. A mouse anti-Myc antibody was used for co-immunostaining with cell-type markers (Figure 4C). For low-magnification assessment of the regional distribution of Myc-tagged ALDH5A1 (Figure S2), coronal sections of the left cerebral hemisphere from untreated *Aldh5a1*^*+/+*^, untreated *Aldh5a1*^−/−^, and mid- and high-dose AAV-treated *Aldh5a1*^−/−^ mice were examined using a rabbit anti-Myc antibody.

### Western blotting analysis

Western blot analyses were used to quantify the restoration of SSADH expression and modulation of neuroglial markers. Brain proteins for ALDH5A1 and Myc were extracted from the right cerebral hemisphere using X-tractor Buffer (Takara Bio, Shiga, Japan) supplemented with a protease inhibitor tablet (cOmplete Mini; Roche, Switzerland) and DNase I (Sigma-Aldrich, St. Louis, MO, USA). For MBP and GFAP analyses, proteins were extracted using TriPrep Protein Extraction Reagent (Sigma-Aldrich, St. Louis, MO, USA). Subsequently, the homogenates were incubated at room temperature for 10 min and centrifuged (12,000 rpm, 30 min, 4°C). The supernatants were collected, and protein concentrations were determined using a TAKARA Bradford Protein Assay Kit (Takara Bio, Shiga, Japan).

Equal amounts of protein were separated on 4%–12% Bis-Tris gels and transferred to polyvinylidene difluoride membranes using an iBlot 3 system (Thermo Fisher Scientific, Waltham, MA, USA). The membranes were blocked and incubated with primary antibodies, followed by appropriate secondary antibodies (Table S5). Proteins were detected using Western BLot Hyper HRP Substrate (Takara Bio, Shiga, Japan), and chemiluminescent signals were captured using an ImageQuant LAS 4000 system (GE Healthcare UK Ltd., Little Chalfont, UK). β-Actin was used as an internal control. For MBP quantification, the signal intensities of all immunoreactive MBP bands were summed for each sample and normalized to the corresponding β-actin signal.

GFAP and MBP were analyzed in mid-dose AAV-treated *Aldh5a1*^−/−^ mice, whereas ALDH5A1 and Myc were assessed in low-dose AAV-treated *Aldh5a1*^−/−^ mice.

These molecular and histological analyses were performed as exploratory assessments of tissue-level effects and were not designed to evaluate dose-response relationships among treatment groups.

### Simultaneous quantification of GABA and GHB using LC-MS/MS

GABA and GHB concentrations in urine were simultaneously quantified using liquid chromatography-tandem mass spectrometry (LC-MS/MS) as biochemical outcome measures. Urine samples (10 μL) were mixed with an equal volume of methanol (10 μL) for protein precipitation and vortexed. The resulting mixture was then diluted 1,000-fold with water containing the internal standards, resulting in an overall 2,000-fold dilution relative to the original urine sample. The internal-standard solution contained 500 ng/mL BHB (2,4-^13^C_2_), 2 ng/mL GABA-d2, and 0.5 ng/mL creatinine-d3. Sample preparation was performed promptly after thawing, and 2 μL of the prepared sample was injected into the LC-MS/MS system.

Analysis was performed using a triple quadrupole LC-MS/MS system (Nexera ×2 UHPLC system coupled to an LCMS-8060 triple quadrupole mass spectrometer; Shimadzu, Kyoto, Japan) operated in multiple reaction monitoring mode. GABA, GHB, creatinine, GABA-d2, and creatinine-d3 were measured in positive electrospray ionization mode, whereas BHB (2,4-^13^C_2_), used as the internal standard for GHB, was measured in negative ion mode. GHB for calibration was synthesized based on a previously described method.^29^

Quantification was based on peak area ratios and calibration curves using LabSolutions LCMS software (v.5.118; Shimadzu, Kyoto, Japan). The calibration ranges were 0.25–100 ng/mL for GABA and creatinine and 10–1,000 ng/mL for GHB. The limits of detection (LOD; signal-to-noise ratio ≥3) were 0.1 ng/mL for GABA and creatinine and 5 ng/mL for GHB, and the lower limits of quantification (LLOQ; signal-to-noise ratio ≥10) were 0.25 ng/mL for GABA and creatinine and 10 ng/mL for GHB. Urinary creatinine was measured simultaneously by LC-MS/MS using creatinine-d3 as the internal standard, and urinary GABA and GHB concentrations were normalized to creatinine levels. GHB samples in which no detectable peak was observed were assigned a value of zero for statistical analysis.

Group comparisons at each time point were performed using one-way ANOVA followed by Tukey’s multiple comparison test. At the 1-week time point, the mid-dose group was excluded from formal statistical comparisons because only a single sample was available. Additional chromatographic conditions, mass spectrometric parameters, and calibration settings are provided in Table S6.

### Statistical analysis

Survival curves were generated using the Kaplan-Meier method and compared with the log-rank (Mantel-Cox) test. HRs and 95% CIs were calculated using the log-rank method implemented in GraphPad Prism. Body weight and rotarod performance over time (Figures 2B and 2D) are presented descriptively as longitudinal data. Because untreated and saline-treated *Aldh5a1*^−/−^ mice exhibited early mortality, resulting in non-random dropout over time, longitudinal statistical comparisons across all time points were not performed. Group comparisons at the selected analysis time points shown in Figures 2C and 2E were performed using one-way analysis of variance (ANOVA) followed by Tukey’s multiple comparison test. Densitometric comparisons of ALDH5A1 and Myc protein expression shown in Figure 4B, GFAP expression shown in Figure 5C, and MBP expression shown in Figure 7H were also performed using one-way ANOVA followed by Tukey’s multiple comparison test. Data are presented as the mean ± standard deviation (SD), unless otherwise indicated. Statistical analyses were performed using GraphPad Prism (v.10; GraphPad Software, San Diego, CA, USA). Statistical significance was set at *p* < 0.05, except for the three pairwise survival comparisons, for which a Bonferroni-corrected threshold of *p* < 0.0167 was applied.

## Data and code availability

The data supporting the findings of this study are available within the article and its supplemental information files.

## Acknowledgments

This work was supported by the JMU Graduate Student Start-up Award, the JMU Graduate Student Research Award, and the JST SPRING Program (grant number JPMJSP2183).

## Author contributions

H.O. conceptualized, supervised the study, and developed the methodology. K.K., T.M., A.H., S.A., and M.W. performed the investigation. K.K. curated the data, conducted the formal analysis, prepared the visualizations, and wrote the original draft of the manuscript. S.-I.M., N.T., and M.I. provided resources. E.F.J., K.M., and S.-I.M. reviewed and edited the manuscript. H.O. and K.K. acquired funding. All authors read and approved the final manuscript.

## Declaration of interests

S.-I.M. has equity with ONODERA GT Pharma, Inc., which commercializes AAV vectors for gene therapy applications.

H.O. and S.-I.M. are inventors on a patent application related to this work (Japanese patent application no. 2026–14709).

## Declaration of generative AI and AI-assisted technologies in the manuscript preparation process

During the preparation of this work, the authors used ChatGPT (OpenAI) to assist with language editing and improvement of English expressions. After using this tool, the authors reviewed and edited the content as needed and take full responsibility for the content of the published article.

## Supplemental information

Document S1. Figures S1–S4 and Tables S1–S6

Document S2. Article plus supplemental information
