# Supplementary material for: AAV vector-mediated ALDH5A1 gene replacement improves survival and metabolic correction in a severe mouse model of SSADH deficiency
Source: Mol Ther Adv. 2026 Oct 7;34(4):201860. doi: 10.1016/j.omta.2026.201860 (PMC13718803; doi:10.1016/j.omta.2026.201860)
Supplement: Supplementary file 1 [file mmc1.pdf]

OMTA, Volume *34*

Supplemental information

AAV vector-mediated *ALDH5A1* gene replacement improves survival and metabolic correction in a severe mouse model of SSADH deficiency

Kiri Koshu, Ayumi Horiguchi, Teru Mizuno, Shiho Aoki, Miyuki Watanabe, Naomi Takino, Mika Ito, Eriko F. Jimbo, Kazuhiro Muramatsu, Shin-Ichi Muramatsu, and Hitoshi Osaka

Supplemental Information

Table S1.

Primer sequences used for PCR genotyping of *Aldh5a1* mice

**Target allele**	Primer	Sequence(5'→3')
Wild-type	Forward	GCTATGACTGTGAACTCTGTCAAGCCAC
Wild-type	Reverse	CATGTTCGCAACTGCCTATAACTCCAGG
Mutant	Forward	GCTATGACTGTGAACTCTGTCAAGCCAC
Mutant	Reverse	AGCGCATGCTCCAGACTGCCTTGG

The PCR comprised 39 cycles at 95℃ for 20sec, 60℃ for 20sec, and 72℃ for 20sec.

Table S2.

Number of animals contributing to longitudinal body weight measurements at each time point.

Age (weeks)	Untreated *Aldh5a1*$^{+/+}$	Untreated *Aldh5a1*$^{-/-}$	Saline *Aldh5a1*$^{-/-}$	High-dose *Aldh5a1*$^{-/-}$	Mid-dose *Aldh5a1*$^{-/-}$	Low-dose *Aldh5a1*$^{-/-}$
1	10	10	11	9	5	0
2	10	14	9	9	6	0
3	10	10	7	9	5	5
4	10	5	4	9	3	1
8	10	0	0	9	3	0
12	10	0	0	7	3	0
16	10	0	0	7	3	0
20	10	0	0	7	3	0
24	10	0	0	6	3	0
28	10	0	0	4	2	0
32	10	0	0	4	2	0
36	10	0	0	4	2	0
40	10	0	0	4	2	0
44	10	0	0	4	2	0
48	10	0	0	4	2	0
52	10	0	0	4	2	0
56	10	0	0	4	2	0
60	10	0	0	4	2	0

Table S3.

Number of animals contributing to longitudinal rotarod measurements at each time point.

Age (weeks)	Untreated *Aldh5a1*$^{+/+}$	Untreated *Aldh5a1*$^{-/-}$	Saline *Aldh5a1*$^{-/-}$	High-dose *Aldh5a1*$^{-/-}$	Mid-dose *Aldh5a1*$^{-/-}$	Low-dose *Aldh5a1*$^{-/-}$
3	10	11	4	9	4	2
4	10	5	4	9	3	0
8	10	0	0	9	3	0
12	10	0	0	7	3	0
16	10	0	0	7	3	0
20	10	0	0	7	3	0
24	10	0	0	6	3	0
28	10	0	0	6	3	0
32	10	0	0	6	3	0
36	10	0	0	6	3	0
40	10	0	0	4	2	0
44	10	0	0	4	2	0
48	10	0	0	4	2	0
52	10	0	0	4	2	0
56	10	0	0	4	2	0
60	10	0	0	4	2	0

Table S4.

The primary and secondary antibodies for immunofluorescence

**Antibody**	**Source**	**Catalog number**	**Dilution**
Chicken anti-β III-tubulin antibody	Abcam	ab107216	1:1000
Rabbit anti-GFAP antibody	Abcam	ab7260	1:500
Rabbit anti-MBP antibody	Proteintech	10458-1-AP	1:100
Rabbit anti-Iba1 antibody	Wako	012-28521	1:200
Mouse anti-Iba1 antibody	Abcam	ab283319	1:200
Mouse anti-Myc antibody	Santa Cruz	9E10, sc-40	1:200
Rabbit anti-Myc antibody	MBL Life Science	562	1:250
Goat anti-rabbit IgG(H+L) Alexa Fluor 488	Thermo Fisher	A11008	1:250
Goat anti-rabbit IgG(H+L) Alexa Fluor 594	Thermo Fisher	A11037	1:250
Goat anti-chicken IgG(H+L) Alexa Fluor 488	Thermo Fisher	A11039	1:250
Goat anti-mouse IgG(H+L) Alexa Fluor 594	Thermo Fisher	A11032	1:250

Table S5.

The primary and secondary antibodies for immunoblotting

**Antibody**	**Source**	**Catalog number**	**Dilution**	**Blocking buffer**
Rabbit anti-ALDH5A1 antibody	Abcam	ab129017	1:10000	5%BSA / TBS-T
Rabbit anti-GFAP antibody	Abcam	ab7260	1:10000	5%BSA / TBS-T
Rat anti-MBP antibody	Selleck	F1098	1:1000	5%skim milk / PBS-T
HRP-conjugated anti-Myc antibody	MBL	M192-7	1:10000	10%skim milk / PBS
HRP-conjugated anti- β -actin antibody	MBL	PM053-7	1:5000	-
mouse anti-rabbit IgG-HRP	Santa Cruz	SC2357	1:1000	
HRP-conjugated Donkey Anti-Rat IgG(H+L)	Proteintech	SA00001-10	1:1000	

Table S6.

LC–MS/MS analytical conditions for quantification of GABA and GHB

Parameter	Condition
LC system	Nexera X2 UHPLC (Shimadzu, Kyoto, Japan)
Mass spectrometer	LCMS-8060 Triple Quadrupole (Shimadzu, Kyoto, Japan)
Column	Discovery HS F5-3, 3.0 μm, 150 × 2.1 mm (Sigma-Aldrich, St. Louis, MO, USA)
Ionization technique	Electrospray ionization (ESI)
Polarity	Positive, except for BHB (2,4-$^{13}C_2$), which was analyzed in negative ion mode
Injection volume	2 μL
Flow rate	0.25 mL/min
Column oven temperature	40 ℃
Autosampler temperature	4 ℃
Mobile phase A	Water with 0.1% formic acid
Mobile phase B	Acetonitrile
Gradient program	2% B (0-1.4 min), linear increase to 30% B (4.0 min), hold at 50% B (5.0-8.0 min), return to 2% B (8.1 min), re-equilibration to 11.0 min
Pump mode	Binary gradient
Ion monitoring mode	Multiple reaction monitoring (MRM)
Heating / drying gas	Nitrogen
Collision gas	Argon
Data acquisition software	LabSolutions LCMS (ver. 5.118, Shimadzu)
Quantification method	Peak area ratio to internal standard
Calibration model	Least-squares linear regression with 1/x weighting
Internal standard solution	BHB (2,4-$^{13}C_2$), 500 ng/mL; GABA-d2, 2 ng/mL; creatinine-d3, 0.5 ng/mL
GABA MRM transition / CE	104.0000 → 87.1000; CE −14
GABA-d2 MRM transition / CE	106.3000 → 89.1000; CE −14
GHB MRM transition / CE	105.0000 → 87.1000; CE −12
BHB (2,4-$^{13}C_2$) MRM transition / CE	105.1922 → 60.0000; CE 14; negative ion mode
Creatinine MRM transition / CE	114.1000 → 44.0500; CE −19
Creatinine-d3 MRM transition / CE	117.1500 → 47.1500; CE −18
Calibration range	GABA and creatinine, 0.25–100 ng/mL<br>GHB, 10–1,000 ng/mL
LOD	GABA, 0.1 ng/mL; GHB, 5 ng/mL; creatinine, 0.1 ng/mL (S/N ≥3)
LLOQ	GABA, 0.25 ng/mL; GHB, 10 ng/mL; creatinine, 0.25 ng/mL (S/N ≥10)

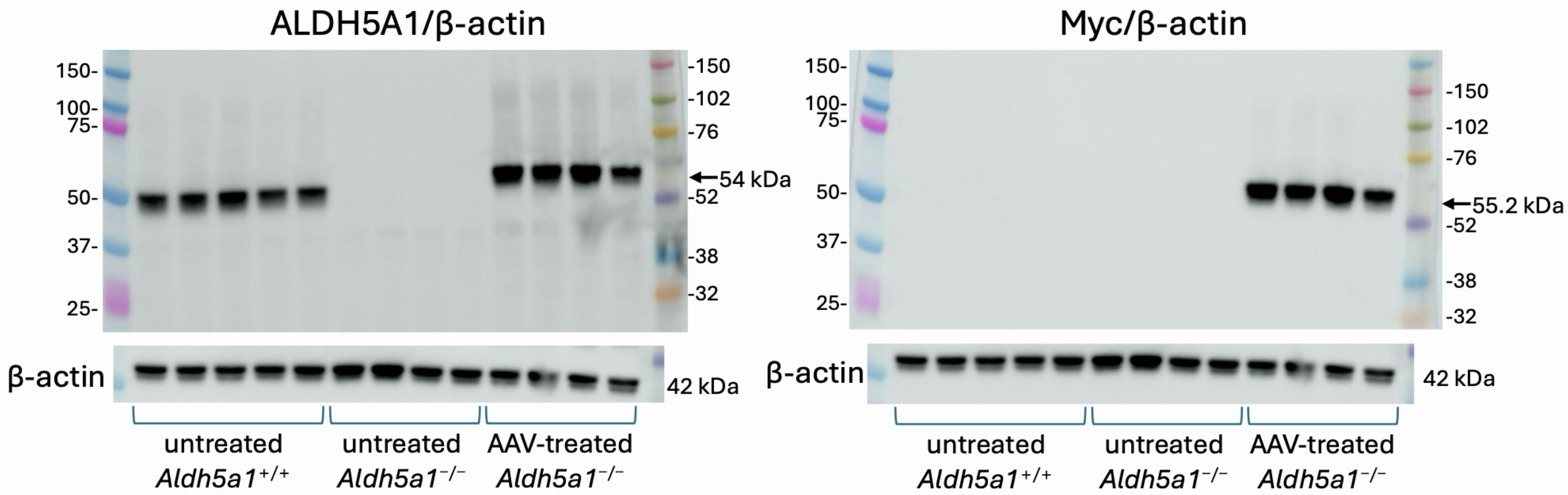

Figure S1. Representative full-length western blot images for aldehyde dehydrogenase 5 family member A1 and Myc.

Representative full-length (uncropped) western blot images corresponding to the quantification illustrated in Figure 4. Right whole-brain lysates from untreated aldehyde dehydrogenase 5 family member A1 (*Aldh5a1*)$^{+/+}$, untreated *Aldh5a1*$^{-/-}$, and adeno-associated virus (AAV)-treated *Aldh5a1*$^{-/-}$ mice are analyzed for total succinic semialdehyde dehydrogenase (SSADH) (detecting endogenous and exogenous proteins) and vector-specific Myc expression. β-actin is used as a loading control.

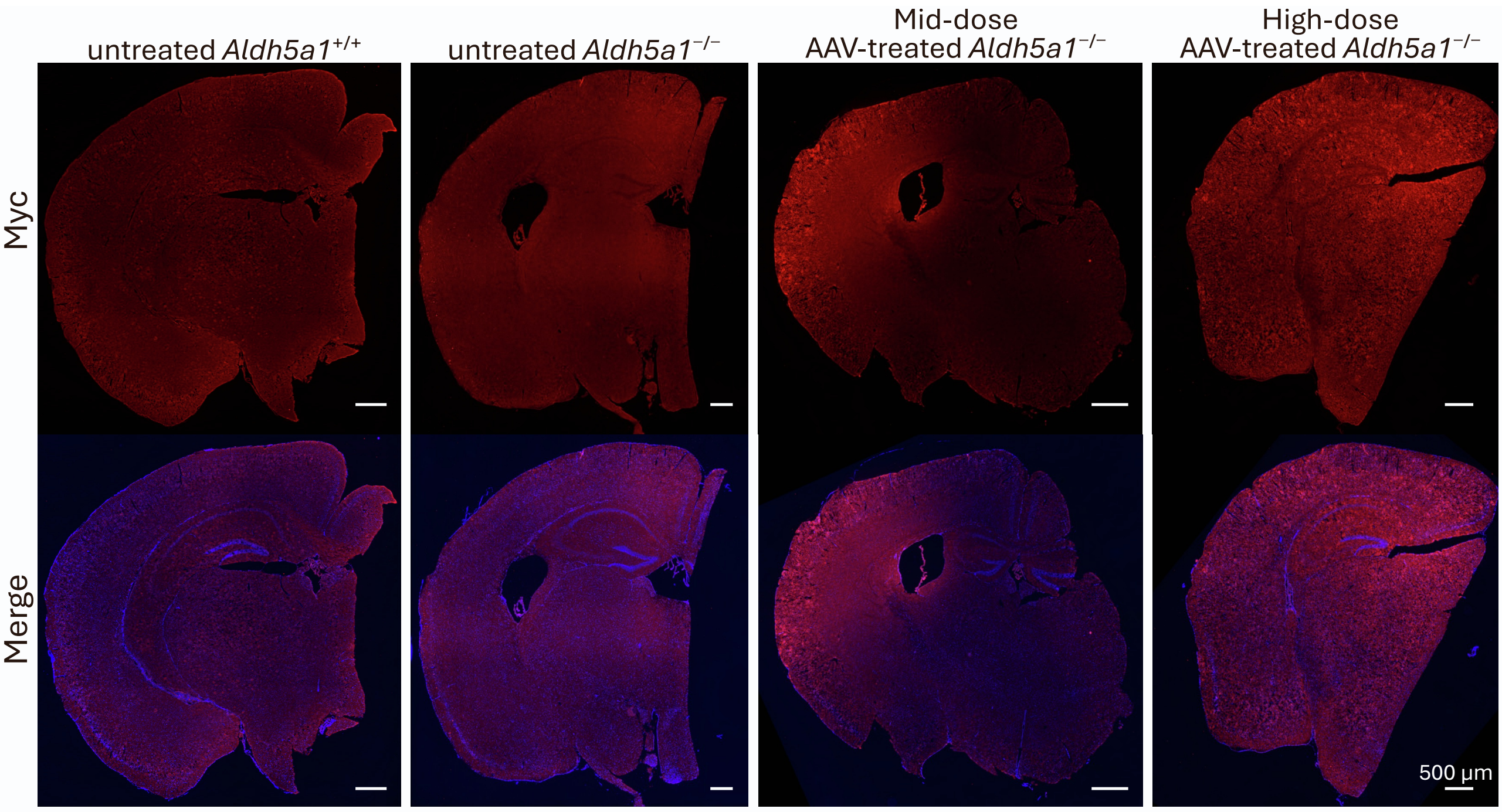

Figure S2. Brain distribution of Myc-tagged aldehyde dehydrogenase 5 family member A1 expression following adeno-associated virus vector treatment.

Representative low-magnification immunofluorescence images of the left cerebral hemisphere from untreated aldehyde dehydrogenase 5 family member A1 (*Aldh5a1*)$^{+/+}$, untreated *Aldh5a1*$^{-/-}$, and mid- or high-dose adeno-associated virus (AAV)-treated *Aldh5a1*$^{-/-}$ mice. Myc immunoreactivity (red) was detected in AAV-treated mice and appeared heterogeneous in the mid-dose section and more widespread in the high-dose section. Nuclei were counterstained with DAPI (blue). Upper panels, Myc; lower panels, Myc/DAPI merge. One mouse per group was examined (n = 1 per group). The mid-dose Myc/DAPI merged image was reused from the low-magnification image shown in Figure 4C; the corresponding Myc-only panel was derived from the same acquisition. These panels are included here to demonstrate regional Myc distribution. Scale bars: 500 µm.

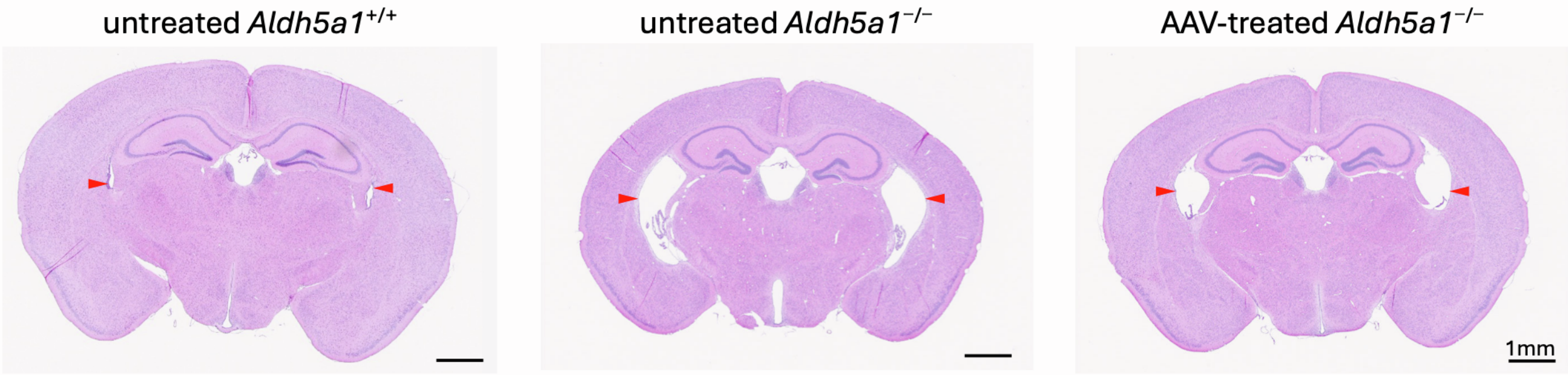

Figure S3. Histopathological assessment of brain morphology following adeno-associated virus vector treatment.
Representative hematoxylin and eosin-stained coronal brain sections obtained from 3-week-old mice. Red arrowheads indicate the lateral ventricles. Untreated aldehyde dehydrogenase 5 family member A1 (*Aldh5a1*)$^{-/-}$ mice exhibited ventricular enlargement and altered hippocampal morphology compared with *Aldh5a1*$^{+/+}$ mice, whereas these abnormalities appeared less pronounced in representative sections from mid-dose adeno-associated virus (AAV)-treated *Aldh5a1*$^{-/-}$ mice. Histological assessment was exploratory in nature and was not intended as a quantitative evaluation of ventricular size or structural recovery. One mouse per group was examined (n = 1 per group). Scale bars: 1 mm.

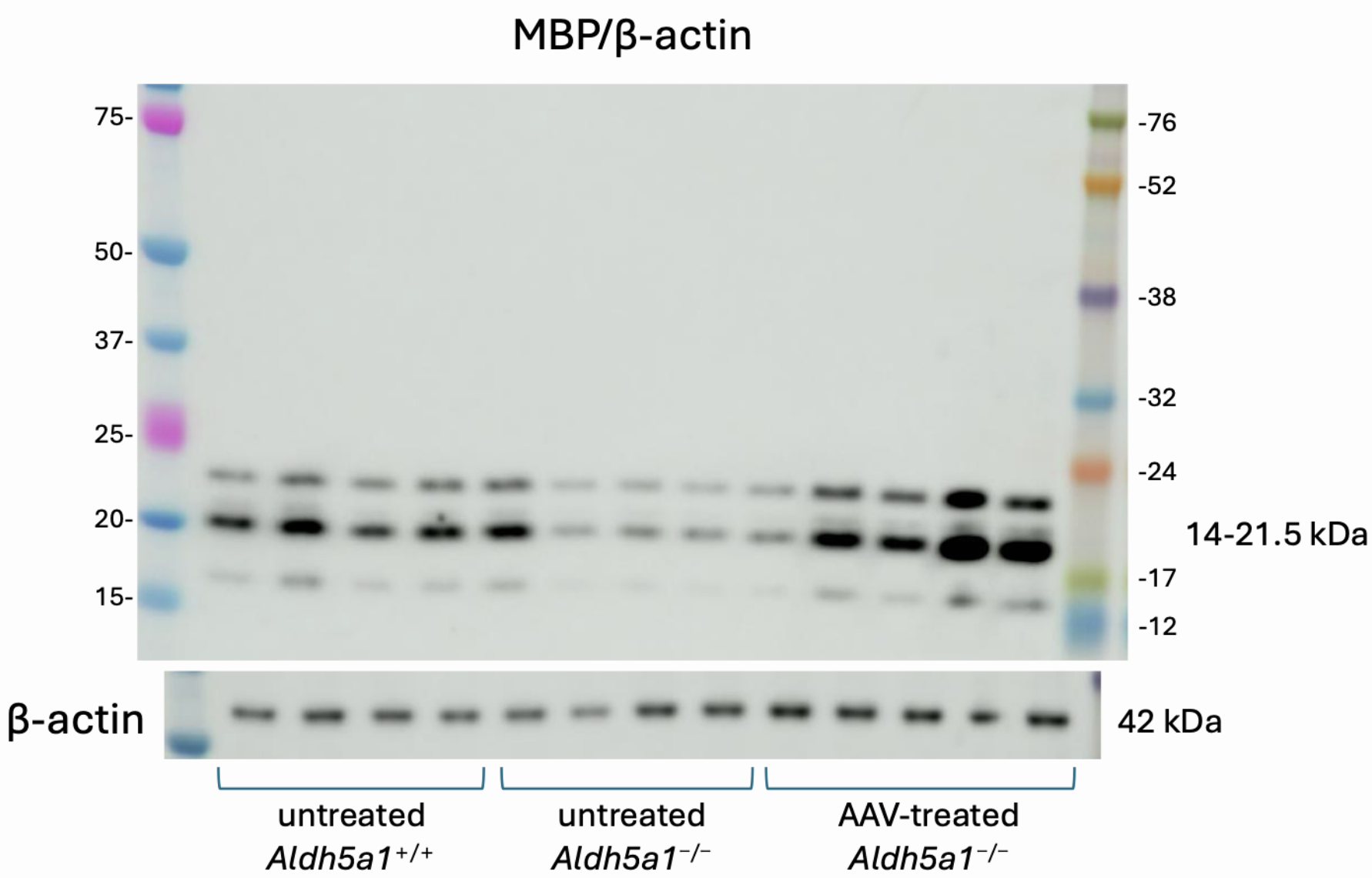

Figure S4. Full western blot images used for myelin basic protein quantification.
Full western blot images showing myelin basic protein (MBP) and β-actin expression in right hemisphere brain lysates from untreated aldehyde dehydrogenase 5 family member A1 (*Aldh5a1*)$^{+/+}$ mice (n = 5), untreated *Aldh5a1*$^{-/-}$ mice (n = 4), and mid-dose adeno-associated virus (AAV)-treated *Aldh5a1*$^{-/-}$ mice (n = 4). The representative blot shown in Figure 7G was selected from these samples. For quantification in Figure 7H, the signal intensities of all immunoreactive MBP bands were summed for each sample and normalized to the corresponding β-actin signal.
